# The (Not So) Changing Man: Dynamic Gender Stereotypes in Sweden

**DOI:** 10.3389/fpsyg.2019.00037

**Published:** 2019-01-30

**Authors:** Marie Gustafsson Sendén, Amanda Klysing, Anna Lindqvist, Emma Aurora Renström

**Affiliations:** ^1^Department of Social Sciences, Södertörn University, Huddinge, Sweden; ^2^Department of Psychology, Stockholm University, Stockholm, Sweden; ^3^Department of Psychology, Lund University, Lund, Sweden; ^4^Department of Psychology, Gothenburg University, Gothenburg, Sweden

**Keywords:** social role theory, gender stereotypes, femininity, masculinity, agency, communion, division of labor

## Abstract

According to *Social Role Theory*, gender stereotypes are dynamic constructs influenced by actual and perceived changes in what roles women and men occupy (Wood and Eagly, 2011). Sweden is ranked as one of the most egalitarian countries in the world, with a strong national equality discourse and a relatively high number of men engaging in traditionally communal roles such as parenting and domestic tasks. This would imply a perceived change toward higher communion among men. Therefore, we investigated the dynamics of gender stereotype content in Sweden with a primary interest in the male stereotype and perceptions of gender equality. In Study 1, participants (*N* = 323) estimated descriptive stereotype content of women and men in Sweden in the past, present, or future. They also estimated gender distribution in occupations and domestic roles for each time-point. Results showed that the female stereotype increased in agentic traits from the past to the present, whereas the male stereotype showed no change in either agentic or communal traits. Furthermore, participants estimated no change in gender stereotypes for the future, and they overestimated how often women and men occupy gender non-traditional roles at present. In Study 2, we controlled for participants’ actual knowledge about role change by either describing women’s increased responsibilities on the job market, or men’s increased responsibility at home (or provided no description). Participants (*N* = 648) were randomized to the three different conditions. Overall, women were perceived to increase in agentic traits, and this change was mediated by perceptions of social role occupation. Men where not perceived to increase in communion but decreased in agency when change focused on women’s increased participation in the labor market. These results indicate that role change among women also influence perceptions of the male stereotype. Altogether, the results indicate that social roles might have stronger influence on perceptions of agency than perceptions of communion, and that communion could be harder to incorporate in the male stereotype.

## Introduction

‘Signs of gender equality are evident everywhere, from men taking their toddlers to preschool in pushchairs every morning to women rising the ranks in traditionally male-dominated industries’ (The Local, 2018).

This quote describes Sweden as an egalitarian country where men are seen in caretaking roles whereas women are seen in typically agentic roles. In fact, Sweden’s national representation and national brand include gender equality as a fundamental part (Towns, 2002; Jezierska and Towns, 2018). Sweden frequently positions itself and is positioned both nationally and internationally as world leading when it comes to gender equality (Towns, 2002). Following social role theory (Eagly and Steffen, 1984; Wood and Eagly, 2011), a result of such perceptions of gender equality in labor division should be that differences in gender stereotype content would decrease.

Agency and communion represent core dimensions of gender stereotype content, where agency is associated with masculine characteristics and communion with feminine characteristics (Abele and Wojciszke, 2014). Agency refers to traits such as independent, assertive and dominant, whereas communion refers to traits such as relationship-oriented, emphatic and caring. Social role theory posits that this division in gender stereotype content is based on observations (in media or in daily life) of women and men in different roles; a division of labor stemming from women’s and men’s differing physical capabilities for child rearing contra labor requiring physical strength (Koenig and Eagly, 2014). When women and men occupy and perform tasks in work and family life, personality traits are derived from behaviors, as described by correspondent inference theory (Gilbert and Malone, 1995). Thus, women are perceived as nurturing and kind because they occupy the majority of caretaking roles (both at home and in the labor market) whereas men are perceived as independent and assertive because they occupy the majority of managerial positions and jobs with higher status (Cejka and Eagly, 1999; Eagly and Wood, 2011). Inspections of job characteristics based on O^∗^Net research by the United States Bureau of Labor Statistics also found positive relationships between communal traits and roles primarily occupied by women, as well as between agentic traits and roles primarily occupied by men (Levanon and Grusky, 2016; Cortes and Pan, 2017). When groups of women or men enter non-traditional roles (i.e., roles requiring characteristics which are not stereotypically associated with that specific gender), social perceivers infer a corresponding shift in personality characteristics to accommodate the new role demands. Evidence of this is that gender stereotypes have been shown to be influenced by perceptions about past, present, and future divisions of labor (Diekman et al., 2005).

So far, the literature on dynamic stereotypes has consistently shown that participants perceive the typical woman of today as more agentic, i.e., having more characteristics associated with masculinity, than the typical woman of previous times (Diekman and Eagly, 2000; Diekman et al., 2005; Wilde and Diekman, 2005; Diekman and Goodfriend, 2006; Garcia-Retamero et al., 2011; Bosak et al., 2017). The perceived change in agency has been quite linear in that masculine characteristics were both perceived to be lower in the past and higher in the future. The shift toward higher perceived agency has been explained by women’s increased participation in the labor market in agentically demanding roles. Accordingly, perceived distribution of women and men in non-traditional roles has been identified as a mediator for perceived changes in gender stereotype content in several studies (Diekman and Eagly, 2000; Diekman et al., 2005; Bosak et al., 2017). Evidence for change in perception of men, in contrast, is not as conclusive. In studies from the United States (Diekman and Eagly, 2000; Diekman et al., 2005) and Germany (Wilde and Diekman, 2005), the perception of men showed no change, in Chile and Brazil (Diekman et al., 2005), masculinity was perceived to increase also in men, whereas in Ghana (Bosak et al., 2017) and Spain (Garcia-Retamero et al., 2011), men were perceived to increase in communality. When results indicated a shift in the perception of men, this was less often mediated by perceived distributions of women and men in non-traditional roles (Bosak et al., 2017).

Furthermore, self-reported data among women and men documented stronger shifts in agency related with social roles than communion (Moskowitz et al., 1994). Diekman and Schneider (2010) consider the interactions between broad gender roles and specific roles, and how they might explain change. For example, if women still do more of the household work that is associated with caregiving, or if they perform more communal tasks at work, they should not be perceived to decrease in communion. Similarly, if men do not work in professions which require communal skills, or enact family roles that are less associated with caregiving, men might not be perceived as acquiring communion only by taking more parental leave.

To our knowledge, past research on dynamic stereotypes has not discussed whether there might be differences in how malleable agentic and communal traits are. For example, perceived gender differences in nurturing are to a greater extent attributed to biological causes than gender differences in math ability (Cole et al., 2007), and motherhood is more strongly related with biology than fatherhood (McPherson et al., 2018). It is therefore possible that communion is seen as a part of a female “essence”, meaning that communal traits may be harder to gain for those not belonging to the category “woman.” However, a recent United States study on the substereotypes of mothers and fathers did find that social perceivers estimated an increase of stereotypical maternal traits in fathers over time, due to fathers being perceived as taking on more maternal tasks (Banchefsky and Park, 2016), meaning that communal traits are possible to include in the stereotype for at least fathers. In comparison to the United States, parental leave is longer in Sweden, and there are special benefits resulting from policies directed toward the non-birth parent. Since these policies have been marketed as an effort to increase parental leave among fathers, paternal roles may be more salient and have higher status in the Swedish society as compared to other countries. The question addressed in the current study is whether changes in parental care among men extend to the general stereotype of men in Sweden, leading to increased perceived communion among Swedish men. Such a shift would occur especially if people see men as more involved in parental care, and if they enact parental roles in the same way as women do (Diekman and Schneider, 2010).

Masculinity, in contrast to femininity have been described as transient, precarious and something that men continuously need to perform (Bosson et al., 2013). Masculinity is also associated with higher status than femininity (Connell and Messerschmidt, 2005; Rudman et al., 2012), indicating that women might benefit from displaying agency, which are some of the trait characterstics of masculinity. Women’s self-ratings of agency (Twenge, 1997) and ratings of women in general (Diekman et al., 2005), have indeed gained in agency over time. However, although women with agentic traits are perceived to be equally competent as men, they may still face social penalties such as being less likeable or hirable compared to men with similar agentic traits and behaviors (Brescoll and Uhlmann, 2008; Rudman et al., 2012; Williams and Tiedens, 2016). Thus, descriptive stereotypes about women might include more agency today than in the past, but prescriptive stereotypes would still require women to avoid excessive agency (Rudman et al., 2012).

Social role theory also acknowledges that contextual factors, such as cultural values, impact inferences from observed role occupation to stereotype content. Cross-cultural research has shown that the male stereotype aligns with the core values of a culture: such that individuals from collectivist cultures rated men as more communal than women, whereas individuals from individualistic cultures rated men as more individualistic than women (Cuddy et al., 2015). Furthermore, research on cultural values has shown that Sweden is rated as individualistic rather than collectivistic (Hofstede, 2001), suggesting that the male stereotype in Sweden would be viewed as containing fewer communal qualities than the female stereotype. However, Sweden is also rated as one of the most feminine countries in the world, meaning that values such as relationships and quality of life are more important than money, objects and work. This indicates that communal roles among men, such as child-rearing, should be valued more highly and of higher status in Sweden as compared to many other countries. In sum, Sweden represents an interesting country in which to investigate if changes in social role occupation can influence the content of both the female and male gender stereotype, because of the strong identification as being a gender egalitarian nation coupled with the presence of individualistic cultural values.

### Sweden and Gender Equality

Sweden does not only have a self-image of being gender equal. In international comparisons Sweden is a highly egalitarian country, being ranked as number five on the Global Gender Gap Index (World Economic Forum, 2017) and as number eight in the Global Leadership and Organizational Behavior Effectiveness (GLOBE) study (Warner, 2012). An international comparison of the parental leave system (Ray et al., 2010) showed that Sweden ranked among the most egalitarian countries when it comes to parental leave among fathers. This ranking can be explained by continuous changes in the social insurance system for parents. The first insurance related to parental leave was introduced in 1954 and referred to as “motherhood insurance.” The implementation of the insurance was intended to motivate families to have more children. At this time, about one third of the women had entered the labor market. The motherhood insurance, together with other family laws, made their position on the labor market less vulnerable (Central Bureau of Statistics [SCB], 1953). In 1974, the term for the insurance changed from “motherhood insurance” to “parental insurance.” At that time, only 0.5% of fathers took any parental leave. Later reforms partly individualized the parental insurance (Warner, 2012), and the first individual month (often called “daddy month” because it was aimed toward fathers taking on more parental leave) was introduced in 1995. The second “daddy month” was introduced in 2002, and the third in 2016 (Statistics Sweden, 2016). Since 1995, fathers have steadily increased their output of parental leave. In 2012, the average of fathers’ leave was 56 days per child (average for mothers was 284 days per child), and 23% stayed at home for more than 3 months (96% of mothers stayed at home for more than 3 months; Inspektionen för socialförsäkringen [ISF], 2012).

Equality in the labor market also has a long history of government interventions. In 1950, 23% of women were active in the labor market, in comparison with 65% of men (Central Bureau of Statistics [SCB], 1953). From the 1950’s, women’s activity in the labor market increased. In 1980, an office of equal opportunities was established as an independent government authority under the Ministry of Labor. The main purpose was to prevent and act against gender discrimination in the labor market and an anti-discrimination law focused on gender equality was enacted at the same time (Law, 1979:1118). Since then, the anti-discrimination law has been expanded and now includes seven grounds for discrimination: gender, transgender identity or expression, ethnicity, religion or other belief, disability, sexual orientation, and age (DA, 208:567). Even though Sweden has a high and gender balanced work force participation from an international perspective, there is still a high degree of gender segregation in terms of actual occupations. In 2016, the work force participation was 84% among women and 89% among men (Statistics Sweden, 2016). In families with children, 82% of the women and 92% of the men worked. More women (29%) than men (11%) worked part time, although this gender difference has decreased over time. In 2005, 45% of the women worked part time, whereas only 6% of the men did. Concerning gender division of labor, only 15–20% of employees work in jobs or industries with an equal gender distribution. In 2010, the Duncan’s D index for occupational segregation (Duncan and Duncan, 1955) indicated that 54% of the Swedish workforce would have to exchange occupations for gender parity to be reached (Halldén, 2014). Among women, 70% work in female-dominated occupations (e.g., nurse, teachers, and receptionist) and among men, 67% work in male-dominated occupations (e.g., drivers, constructions workers, managers; Warner, 2012). Furthermore, the vertical segregation between women and men is larger in Sweden than in many other European countries (Ellingsæter, 2014). Women leaders are common in the public sector and in politics (50%), fewer in the private sector (30%), and very few among stock listed companies (CEO:s = 5%; Statistics Sweden, 2016).

Applying social role theory to a Swedish context makes a few issues visible. For example, although the labor force participation of women is high, possibly leading to higher ratings of agency of women, women are still primarily working in occupations which require a high degree of communion (Cejka and Eagly, 1999). Furthermore, although Swedish men take more parental leave than elsewhere nowadays (Ray et al., 2010), they do not take out as much as Swedish women do, nor have they entered into communally demanding occupations (Statistics Sweden, 2016).

In Sweden, compared to other countries, another complicating factor might be a mismatch between the high ranking on gender equality scales (Warner, 2012), the discourse reported in the media (Towns, 2002), and actual gender labor division (Statistics Sweden, 2016). The discourse and gender equality rankings might lead to the notion that sufficient gender equality has been reached, making future change both unnecessary and impossible and therefore not expected. It might also lead to overestimations of women’s and men’s non-traditional role performance. If mismatches in perceptions from different sources influence stereotype content and gender distributions in social roles, this indicates a missing piece of the puzzle between social perceivers’ observations and gender stereotype content. It is therefore important to determine whether stereotype content derives from estimates of women and men in different roles based on actual observation of role occupation, which indicates that there is still room for improvement in the future, or from general perceptions of gender equality, and that gender equality has been reached.

## Overview of the Current Research

To investigate how social change in Sweden influences perceptions of women and men of the past, present, and future, we asked participants to rate an average Swedish woman or man of these three time points. This design aligns with the social role theory paradigm previously used to examine dynamic stereotypes (Diekman et al., 2005; Diekman and Goodfriend, 2006). In Study 1, stereotype content was measured for women and men at all three time points. Because of the strong gender equality discourse in Sweden, we expected that participants would indicate a change in traits from the past to the present but not from the present to the future. We expected a change in agentic traits for women and a change in communal traits for men.

We also tested whether participants’ estimates of labor distribution align with official statistics at present time, and if these estimates can explain the changes in stereotype contents. As in previous studies (e.g., Diekman and Eagly, 2000; Wilde and Diekman, 2005; Bosak et al., 2017), perceived non-traditionalism, i.e., the number of individuals in gender counterstereotypical social roles, is tested as a mediator for change in gender stereotype content over time. More specifically, the perception of women’s higher agency should be mediated by non-traditionalism in male-dominated roles, whereas the perception of men’s higher communion should be mediated by non-traditionalism in female-dominated roles. This moderated mediation is suggested because an increase in counterstereotypical roles should be associated with an increase of the characteristics associated with those roles among the gender that is perceived to change; but not among the gender that is not perceived to change. As shown in past studies, mediation effects might be stronger for agency than communion (Bosak et al., 2017). This division into agentic and communal non-traditionalism provides a direct test of the social role theory hypothesis that characteristics associated with specific roles increase corresponding characteristics in those performing the roles.

In Study 2, we investigated if controlling for the participants’ knowledge of objective change in women’s or men’s roles influenced perceptions of non-traditionalism in occupational and domestic roles as well as stereotype content. We presented participants with information regarding the actual change from the past to the present in social role occupation, either focusing on role change for women or men. By this design, we directly compare if changes in communal and agentic tasks lead to similar perceived changes in stereotype content from the past to the present.

Both studies were carried out in accordance with national guidelines on ethical research (Swedish Research Council, 2017). This means that participants were informed about their voluntary and anonymous contribution, and that they could quit the survey whenever they wanted without giving any reasons for quitting. They were also informed that results would be presented on aggregated levels with no possibility to extract any personal information. After this information, participants gave their informed consent and were electronically forwarded to the questionnaire. After answering the questionnaire, participants actively submitted their responses. A formal ethical approval is not mandatory for this type of research because it did not include any biodata nor did it intend to affect the participants physically or psychologically. It also did not entail any handling of sensitive data as described in the Swedish law about personal data.

## Study 1

The main purpose of Study 1 was to provide results directly comparable to previous research on dynamic gender stereotype content. Hence, Study 1 used the same design as has been used in previous research from other countries (e.g., Diekman and Eagly, 2000; Wilde and Diekman, 2005; Bosak et al., 2017) to establish the content of gender stereotypes of women and men of the past, present, and the future.

### Materials and Methods

#### Participants and Design

Participants (*N* = 399, *M*_age_ = 48.87, *SD*_age_ = 18.01) were recruited from an existing web panel consisting of 67,000 individuals. Stratification was performed on the web panel participants based on gender, age (in 10-year intervals) and geographic region and participants were randomly selected for participation within quotas that were representative of the Swedish population. Of the 399 participants starting the survey, 323 participants completed it (response rate = 80.95%). Participants indicated their gender with a free text response (women = 51.39%, men = 45.55%, non-binary = 2.17%, did not indicate gender = 0.93%).

The design was a 2 (target gender) × 3 (time) between-subjects factorial design with personality, cognitive and physical characteristics as outcome measures. Participants were randomized to conditions in which they evaluated either a woman or a man in the past (year 1950), the present (year 2017), or the future (year 2090). Because the current analysis plan includes a somewhat large amount of multiple testing, we calculated the false discovery rate (FDR) for Study 1 *ad hoc*. We made the decision to use FDR instead of a more conservative alpha correction in order to retain statistical power and give an intuitively informative coefficient (Benjamini and Hochberg, 1995). The FDR was calculated with the Benjamini–Hochberg method using the sgof package version 2.3 (Castro-Conde and de Uña-Álvarez, 2014) in R version 3.5.1. The total FDR for Study 1 was 1.92% which suggests that the overall risk of falsely rejecting the null hypothesis was under 5%.

### Measures

#### Perceived Role Non-traditionalism in Agentic and Communal Roles

Participants estimated the percentage of the counterstereotypic gender within either traditionally female- or male-dominated occupational and domestic roles (e.g., Diekman and Eagly, 2000; Steinmetz et al., 2014). The occupations were selected from official Swedish labor statistics (Statistics Sweden, 2016), had a minimum of 75% gender homogeneity and should be well-known occupations to the public. The domestic roles were based on official statistics regarding time spent on household tasks in Sweden (Statistics Sweden, 2012), and on items used in previous studies on social role theory (e.g., Diekman and Eagly, 2000). Agentic non-traditionalism (α = 0.87) included estimates of women in male-dominated occupations (*car mechanic*, *pilot, civil engineer*, and *stock broker)* and domestic tasks typically performed by men *(car repairs, paying household bills, changing light bulbs, solving technology problems*, and *doing home repairs*). Communal non-traditionalism (α = 0.85) included estimates of men in female-dominated occupations (*pre*-*school teacher, receptionist*, and *nurse)* and domestic tasks typically performed by women (*doing the laundry, cooking, cleaning, playing with children, assisting children with homework*, *taking care of sick children*, and *caring for children’s appearance*)^1^.

#### Gender Stereotype Dimensions^2^

Participants evaluated 30 characteristics representing traits that are typically associated with femininity or masculinity (Cejka and Eagly, 1999). Both positive and negative items were used (Diekman and Eagly, 2000). Because these characteristics were chosen to include both positive and negative characteristics associated more strongly with either women or men, rather than communion and agency more broadly, we will use the terms femininity and masculinity: even though the positive femininity and masculinity subscale do correspond to the constructs of communion and agency, respectively. Each characteristic was evaluated on a scale from 1 (*not at all likely*) to 7 (*very likely*). Internal reliabilities for final scales were^3^: positive masculinity (α = 0.76), negative masculinity (α = 91), positive femininity (α = 89), and negative femininity (α = 74). See Appendix A, Table A1 for all items used in the scales and Swedish wording.

### Results

Because past research has shown strongest results for personality characteristics, and because cognitive and physical characteristics showed very few significant differences, we chose to streamline this paper and focus on personality characteristics. Results for the cognitive and physical dimensions can be found in Appendix B, Tables B1, B2. Mediation analyses for these dimensions can be found in Appendix C.

Analyses of variance (ANOVAs) are reported for each dependent variable separately. The presence of moderated mediation was determined using an index of moderated mediation (Hayes, 2015). Throughout this article, *p*-values of 0.05 or less are considered as significant. Because participants’ gender did not interact with stereotype content in any consistent pattern, these analyses are omitted.

#### Perceived Role Non-traditionalism

To test participants’ perceptions of agentic and communal non-traditionalism over time, we conducted a 3 (year) × 2 (agentic/communal non-traditionalism) mixed ANOVA with agentic and communal non-traditionalism as within-subjects factors and year as between-subjects factor. A significant main effect of time, *F*(2,313) = 194.34, *p* < 0.001, $\eta_{p}^{2}$ = 0.55, revealed that non-traditionalism increased from the past to the future (*p* < 0.001), whereas the present did not differ from the future (*p* = 0.80; see Table 1). There was neither a significant effect of type of non-traditionalism, *F*(1,313) = 0.83, *p* = 0.36, $\eta_{p}^{2}$ < 0.01, nor a significant interaction with time, *F*(2,313) = 1.32, *p* = 0.27, $\eta_{p}^{2}$ = 0.01. Thus, Swedish participants believed that the past was more traditional in terms of gendered division of labor than the present time. They also estimated similar changes for communal and agentic non-traditionalism from the past to the present.

**Table 1:** Study 1: Means and standard deviations by year for role non-traditionalism.

	Communal role non-traditionalism		Agentic role non-traditionalism	
Target year	*M*	*SD*	*M*	*SD*
1950	13.38_a_	12.34	14.22_a_	13.84
2017	34.23_b_	8.97	33.49_b_	9.87
2090	34.25_b_	9.30	32.05_b_	9.52


*Ratings of role non-traditionalism were made through estimating percentages (0–100) of women and men occupying social roles. Means with different subscripts across time points differ significantly at p < 0.05.*

However, participants did not expect any further change in the future. This could be explained by an overestimation of non-traditionalism at present times. Participants estimated higher non-traditionalism than actual distributions in all gender-typical occupations (see Table 2). In Table 3, we also present percentages on estimated division of domestic duties, although these data cannot be compared to any official statistics.

**Table 2:** Study 1: Mean estimates of percentages of women and men working in different occupations compared to official labor statistics (Statistics Sweden, 2016).

Gender division in occupations				

	Participant estimates		Statistics Sweden statistics	
Occupations	Women	Men	Women	Men
Car mechanic	20.32	69.80	2.98	97.02
Pilot	25.30	66.53	7.19	92.81
Civil engineer	37.06	54.26	16.01	83.99
Stock broker	31.48	60.87	23.62	76.38
Pre-school teacher	62.02	25.95	95.66	4.34
Hair dresser	54.08	31.00	87.54	12.46
Receptionist	62.11	25.64	80.14	19.86
Nurse	58.48	29.18	89.52	10.48
Salesperson	51.02	36.19	63.58	36.42
Physician	42.79	48.96	53.19	46.81
Journalist	43.70	44.81	52.14	47.86
University teacher	41.54	48.91	45.77	54.23


**Table 3:** Study 1: Estimated percentage of household tasks performed by the woman in a heterosexual household with children by year.

	Time					
	1950		Today		2090	
Household task	*M*	*SD*	*M*	*SD*	*M*	*SD*
Car repairs	4.45	13.78	23.01	15.55	23.54	14.04
Changing lightbulbs	18.32	20.65	39.92	17.53	40.32	17.86
Home repairs	14.30	16.43	32.65	14.86	31.65	16.47
Solving technology problems	10.45	14.86	34.00	15.84	32.33	17.25
Paying bills	25.29	27.14	48.43	12.92	47.74	13.14
Cleaning	93.64	12.41	65.53	14.02	65.38	13.27
Laundry	95.31	11.69	65.62	14.91	66.37	13.91
Cooking	93.43	12.57	57.58	11.93	58.27	13.17
Playing with children	79.29	18.12	59.49	12.75	58.47	13.07
Assisting children with homework	76.76	22.31	57.75	11.36	56.41	11.49
Caring of sick children	93.20	15.06	65.87	13.82	64.37	16.15
Caring of children’s appearance	93.22	12.27	67.52	14.98	67.86	13.13


*Ratings of performance of household tasks were made through estimating percentage of task performed by either the woman or the man in a heterosexual relationship: 0 = task only performed by man, 100 = task only performed by woman*.

#### Gender Stereotype Content

Descriptive data for gender stereotypical personality dimensions are presented in Tables 4, 5. Four 2 (Target gender) × 3 (Year) between-subjects ANOVAs were computed to test the effect of time and target gender on gender stereotypical characteristics; *p*-values for all pairwise comparisons were corrected using Tukey’s HSD with a family-wise error rate of 0.05. The personality dimensions were (1) positive femininity, (2) negative femininity, (3) positive masculinity, and (4) negative masculinity.

**Table 4:** Study 1: Means and standard deviations for masculine personality, over time and target gender.

	Positive	Negative
Target gender and year	*M (SD)*	*M (SD)*
**Women**		
1950	3.26_a1_ (0.88)	2.42_a1_ (0.75)
2017	4.20_b1_ (0.94)	3.62_b1_ (1.12)
2090	4.01_b1_ (0.72)	3.38_b1_ (0.90)
Total	3.81 (0.94)	3.13 (1.06)
**Men**		
1950	3.89_a2_ (1.00)	3.38_a2_ (0.99)
2017	4.16_a1_ (0.65)	3.91_al_ (1.10)
2090	4.12_a1_ (0.80)	4.02_ab1_ (1.12)
Total	4.06 (0.83)	3.78 (1.11)


*Within each target gender, means with different subscripts (a,b) differ significantly at p < 0.05 between time points. Within each time point, means with different subscripts (1,2) differ significantly at p < 0.05 between women and men. Ratings were on a 7-point scale on which higher numbers indicate greater likelihood of possessing the characteristics*.

**Table 5:** Study 1: Means and standard deviations for feminine personality, over time and target gender.

	Positive	Negative
Target gender and year	*M (SD)*	*M (SD)*
**Women**		
1950	5.03_a1_ (0.92)	3.50_a1_ (0.81)
2017	4.58_a1_ (0.94)	4.03_b1_ (1.08)
2090	4.58_a1_ (0.70)	3.76_ab1_ (0.87)
Total	4.73 (0.88)	3.76 (0.94)
**Men**		
1950	3.83_a2_ (0.88)	3.47_a1_ (0.91)
2017	3.95_a2_ (0.87)	3.77_ab1_ (0.87)
2090	3.94_a2_ (0.91)	3.86_b1_ (0.98)
Total	3.91 (0.89)	3.71 (0.94)


*Within each target gender, means with different subscripts (a,b) differ significantly at p < 0.05 between time points. Within each time point, means with different subscripts (1,2) differ significantly at p < 0.05 between women and men. Ratings were on a 7-point scale on which higher numbers indicate greater likelihood of possessing the characteristics*.

There was a difference between women and men in stereotype congruent directions for three personality dimensions: positive femininity and positive and negative masculinity (*p*’s < 0.05); whereas women and men did not differ in negative femininity (*p* = 0.54). Three personality dimensions (negative femininity, positive and negative masculinity) were believed to increase over time (*p*’s < 0.05), whereas positive femininity did not differ across time points (*p* = 0.25). The main effects of target gender were qualified by interactions with time for masculinity, but not for positive femininity.

#### Masculinity

The interaction of Target Gender × Year was significant for positive masculinity, *F*(2,317) = 4.41, *p* = 0.01, $\eta_{p}^{2}$ = 0.03, and marginally significant for negative masculinity, *F*(2,317) = 2.90, *p* = 0.06, $\eta_{p}^{2}$ = 0.02. For positive masculinity, pairwise comparisons showed an increase for women between the past and the present (*p* < 0.001), but not between the present and the future (*p* = 0.86). There was no perceived change for men between the past and the present (*p* = 0.55) or between the present and the future (*p* = 1.00). In addition, women and men were rated equally in the present (*p* = 1.00) and the future (*p* = 0.98), whereas they were rated as differing in the past (*p* < 0.01). For negative masculinity, both women and men increased from the past to the present (*p*’s < 0.05) whereas there was no perceived change from the present to the future (*p*’s > 0.81). A simple effects analysis showed a larger increase for women, *F*(2,317) = 21.42, *p* < 0.001, $\eta_{p}^{2}$ = 0.19, than men, *F*(2,317) = 6.10, *p* < 0.01, $\eta_{p}^{2}$ = 0.04. In addition, women and men were rated equally on negative masculinity in the present time (*p* = 0.70), and the future (*p* = 0.07), but differing in the past (*p* < 0.001).

#### Femininity

We expected a perceived change in feminine traits among men but not women, and although a significant interaction for positive femininity was found, *F*(2,317) = 3.71, *p* = 0.03, $\eta_{p}^{2}$ = 0.02, pairwise comparisons showed no significant perceived change among men from the past to the present (*p* = 0.99) or from the present to the future (*p* = 1.00), nor among women (*p* = 0.09 and 1.00, respectively). Across all time points, women were rated higher on femininity than men (*p* < 0.01). For negative femininity, there was no significant interaction *F*(2,317) = 0.98, *p* = 0.38, $\eta_{p}^{2}$ = 0.01.

**FIGURE 1:**
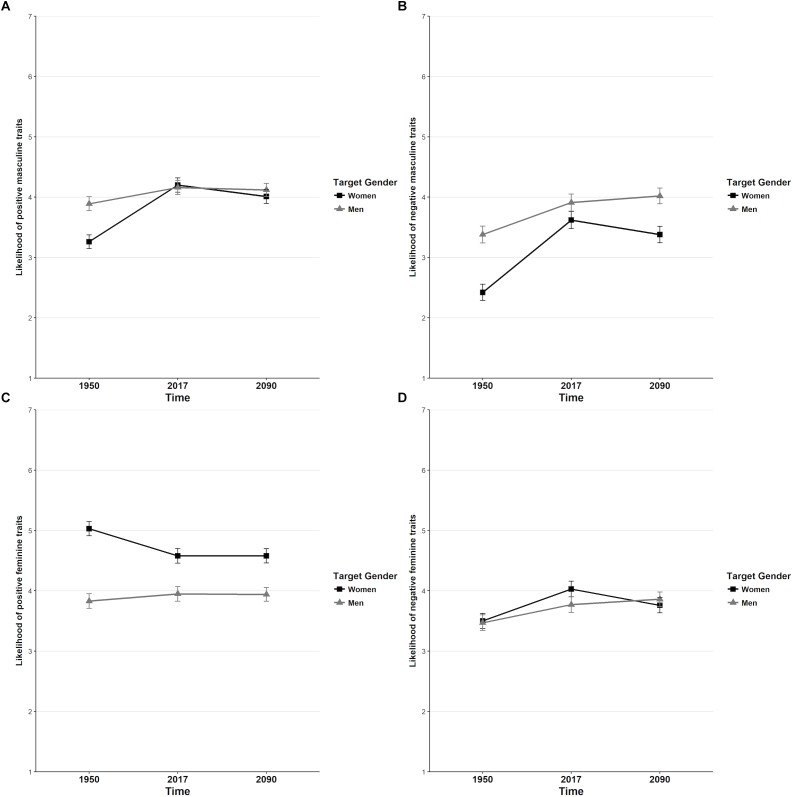
**(A)** change in positive masculine characteristics over time, **(B)** change in negative masculine characteristics over time, **(C)** change in positive feminine characteristics over time, **(D)** change in negative feminine characteristics over time. Error bars represent standard errors of the means.

Thus, for masculinity the ratings aligned with expectations, but not for femininity. Swedish participants also believed that women and men have equal degrees of masculinity in present time, and that women are still more feminine than men. See Figure 1 for a visualization of perceived stereotype content change over time.

#### Correspondence Between Roles and Gender Stereotypes

We used moderated mediation analyses to test if perceived changes in gender stereotype content over time was mediated by increased non-traditionalism in corresponding social roles, and if this mediation was moderated by gender. More specifically, an increase of women in male-dominated roles (agentic non-traditionalism) should lead to an increase in women’s perceived masculinity but not a decrease in men’s perceived masculinity, whereas an increase of men in female-dominated roles (communal non-traditionalism) would lead to an increase in men’s perceived femininity but not a decrease in women’s perceived femininity. To control for an effect of perceived general change over time, the direct effect of year moderated by target gender was also included in the statistical model.

**FIGURE 2:**
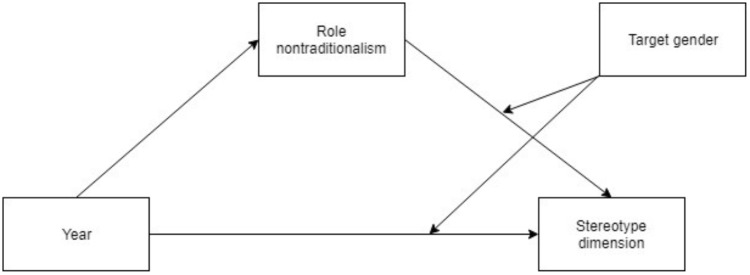
A second stage moderated mediation model with the direct path allowed to be conditional on the moderator.

The moderated mediation models were tested for all stereotype dimensions independent of direct effects of time. The decision to conduct mediation analysis on estimates of femininity despite an absence of a total effect of time was made due to the possibility of a completely indirect effect (for a discussion on completely indirect effects, see Hayes, 2009). The SPSS macro PROCESS, v. 3.00, model 15 (Hayes, 2018) was used to perform the moderated mediation analyses with 95% confidence intervals calculated using a percentile bootstrap approach with 10 000 bootstrap samples. Percentile bootstrapping was chosen because it has been shown to retain the increased power for testing mediation which bootstrapping methods provide, and having only a slightly elevated Type I error rate compared to the inflation of the Type I error rate that comparable bootstrapping methods entails (Fritz et al., 2012). The presence of a moderated mediation effect was determined using an index of moderated mediation (Hayes, 2015). Target gender was dummy coded (0 = “woman,” 1 = “man”), and time was contrast coded such that a one-unit increase represents a perceived change from past (-1) to present (0), and from present to future (1), since the time distance between the conditions was ordered and roughly equivalent. See Figure 2 for a visualization of the statistical model.

For positive masculinity, the index of moderated mediation showed an indirect effect of time through agentic role non-traditionalism moderated by target gender, indicating that the effect of time was mediated for women targets, *b* = 0.24, *SE* = 0.08, *LLCI* = 0.11, *ULCI* = 0.42, but not for men targets, *b* = 0.02, *SE* = 0.05, *LLCI* = -0.06, *ULCI* = 0.12. There was no significant direct effect of time, nor was there a direct effect moderated by target gender. Women’s increase in positive masculinity over time was thus completely qualified by their increased numbers in agentically demanding roles over time.

For negative masculinity, the index of moderated mediation showed an indirect effect of time through agentic role non-traditionalism moderated by target gender. The effect of time was mediated for perception of women targets, *b* = 0.16, *SE* = 0.07, *LLCI* = 0.04, *ULCI* = 0.30, but not for perception of men targets, *b* = -0.02, *SE* = 0.06, *LLCI* = -0.16, *ULCI* = 0.08. There was also a significant direct effect of time which was not moderated by target gender, *b* = 0.32, *SE* = 0.12, *LLCI* = 0.09, *ULCI* = 0.55. Women’s increase in negative masculinity, but not men’s, was partially an effect of increased occupancy of agentically demanding roles, there was also a general increase in these characteristics over time for both women and men independently of social role occupation. See Table 6 for path coefficients and indexes of moderated mediation for both models.

**Table 6:** Study 1: Unstandardized regression coefficients (standard errors in parentheses) with confidence intervals for estimating the indirect conditional effect of time on masculine personality through agentic non-traditionalism, moderated by target gender.

	Agentic role non-traditionalism		Masculine personality positive		Masculine personality negative	
Predictors	*b*	95% *CI*	*b*	95% *CI*	*b*	95% *CI*
Time	8.69^∗∗∗^ (0.83)	7.05, 10.32	0.13 (0.10)	–0.06, 0.32	0.32^∗∗^ (0.12)	0.09, 0.55
Agentic role non-traditionalism			0.03^∗∗∗^ (0.01)	0.02, 0.04	0.02^∗^ (0.01)	0.003, 0.03
Gender			0.83^∗∗∗^ (0.23)	0.38, 1.28	1,14^∗∗∗^ (0.28)	0.58, 1.69
Time × Gender			–0.03 (0.13)	–0.29, 0.23	0.02 (0.16)	–0.30, 0.35
Agentic role non-traditionalism × Gender			–0.03^∗∗^ (0.01)	–0.04, –0.01	–0.02^∗^ (0.01)	–0.04, –0.001
Constant	26.45^∗∗∗^ (0.69)	25.10, 27.80	3.15^∗∗∗^ (0.17)	2.82, 3.47	2.64^∗∗∗^ (0.22)	2.22, 3.06
	*R^2^* = 0.26		*R^2^* = 0.13		*R^2^* = 0.18	
	*F*(1,314) = 109.38, *p* < 0.001		*F*(5,310) = 9.47, *p* < 0.001		*F*(5,310) = 13.33, *p* < 0.001	
Index of moderated mediation			Index = –0.22, 95% CI = –0.41, –0.06		Index = –0.18, 95% CI = –0.38, –0.03	


*^∗^*p* < 0.05, ^∗∗^*p* < 0.01, ^∗∗∗^*p* < 0.001.*

Furthermore, to test that communal non-traditionalism did not affect masculinity, identical moderated mediation models for non-traditionalism in communal roles were tested on masculinity (see Appendix C, Table C1). Results showed that perceived changes in communal roles did not have any effect on perceived masculinity, neither direct nor indirect. Consequently, the increase in perceived masculinity among women over time was a result of higher perceived role non-traditionalism in agentic roles.

For femininity, we found no significant mediation of communal nor agentic non-traditionalism, nor an indirect effect conditional on gender (see Appendix C, Tables C2, C3 for analysis details); meaning that no support was found for a completely indirect effect of time on femininity through communal non-traditionalism conditional on gender.

### Discussion

Study 1 showed that Swedish participants perceived gender stereotypes as dynamic constructs from the past to the present but not from the present to the future, at least in regards to the female stereotype. Women were perceived as more agentic today than in the past, whereas perception of men did not differ based on time. Furthermore, Swedish participants did not expect any change in division of labor in the future. The lack of expected future change differs from past studies on dynamic stereotypes and could indicate an opinion among Swedish people that gender equality has already been reached and that no further change is expected or considered necessary. This interpretation is supported by the strong overestimation of gender balance in occupations which are actually strongly gender segregated. That Swedish participants have a “mental image” of Sweden as a more egalitarian country than it is was indicated both by the estimates of non-traditionalism and by the non-existent change in the future. Participants also rated that women and men had converged on positive and negative masculinity, and that they never differed on negative femininity. The only difference in 2017 was on positive femininity where women were perceived as more communal than men, which could indicate that communal traits are more difficult to gain for men, that changes in men’s parental care are too small, or that men enact parental care with less communion than women do.

From the past to the present, Swedish participants believed that both women and men increased their participation in non-traditional roles. However, this role-change only mediated perceptions of women, meaning that the increase in masculinity was explained by an increase of women in agentically demanding social roles. The perception of men, in contrast, did not change from the past to the present, despite a perceived increase of men in social roles requiring communal behavior. Interestingly, participants strongly, but falsely, believed that men have entered female-dominated roles, which would imply a perceived change also in traits. However, no such relationship between men’s entry into communal roles and a perceived increase in femininity in the male stereotype was found. This indicates that the mechanisms behind perceived stereotype change might operate differently for femininity/communion and masculinity/agency or for perceptions of women and men. One explanation might be that the communal traits are seen as more essential, whereas agentic traits are seen as more strongly related to behavior. Theories about precarious manhood(Bosson et al., 2013) have shown that masculinity is something that men need to perform and establish over time, whereas femininity is seen as a natural consequence of being born as a woman. Femininity is hence not seen as something that women need to perform to the same degree – but seen an essential aspect of being a woman. Women can also gain status through increased displays of traits associated with masculinity, whereas avoidance of femininity is important for men’s maintenance of masculinity.

By using moderated mediation analyses, we showed that women’s perceived increase in masculine traits was specifically associated with a perceived change in women’s agentic roles and not associated with any perceived change among men or in communal roles, which is a strong test supporting social role theory. However, the related analyses to test the mediation of men’s feminine characteristics by change in men’s communal roles were not significant; which indicates that a different mechanism than correspondence inference may be responsible for determining male stereotype content. Similar patterns of full mediation for women, but lack of mediation for men, have been found before (see for example Bosak et al., 2017). Because of the strong “gender equality” discourse in Sweden, we suspect that participants’ estimates of division of labor was based more on an “egalitarian bias” than an actual reflection of role change. To control for such effects, we performed a second study where participants were presented with information about the actual changes in the gendered division of labor roles over time.

## Study 2

In Study 2, participants were presented with factual descriptions of how gender equality in social role occupation increased in Sweden from the 1970s until today. We framed the role change to focus on either women or men to test whether a focus on women’s increase in agentic roles or men’s increase in communal roles influenced perceptions of femininity and masculinity, respectively. Following the results in Study 1 showing that femininity might be more difficult to associate with men than masculinity with women, we believed that explicitly presenting how men’s participation in domestic and parental tasks have increased over time would lead to an increase in femininity but that a control condition or a condition that describes women’s increased participation in the labor market would not.

### Materials and Methods

#### Participants and Design

Participants were recruited from web forums on social media pages focused on student forums recruiting participants to psychological research and a student participant pool hosted by Gothenburg University. A total of 676 participants completed the survey, 28 participants were removed from the experimental conditions for failing to answer control questions correctly. The final sample consisted of 648 participants (women = 74.23%, men = 23.92%, non-binary = 1.08%; *M*_age_ = 25.58, *SD*_age_ = 9.72).

We used a 3 (Framing of Role Change: women’s increase in agentic roles/men’s increase in communal roles/control group) × 2 (Target Gender: women/men) × 2 (Year: 1950/2017) between-subjects factorial design. Participants were randomized to one of the conditions where they read either about women’s change in agentic roles, men’s change in communal roles or to a control condition, and rated either a typical woman or a typical man of the past (1950) or the present (2017). Since we found no change from the present to the future in Study 1, only the past and the present were included in this study. The false discovery rate (FDR) for Study 2 was calculated in the same way as for Study 1. The total FDR for Study 2 was 2.20% which suggests that the overall risk of falsely rejecting the null hypothesis was under 5%.

### Measurement Instruments

#### Framing of Role Change

Two texts were created which described an actual change in division of labor for women or men and titled “Women take more responsibility in the labor market” and “Men take more responsibility in the home.” The text about women focused on changes in women’s participation in the labor market since the mid-1900s (e.g., increasing participation in paid labor and entry into professions previously dominated by men). A graph illustrated the change in employment rate of women and men from 1970 to 2018. The text about men focused on changes in men’s participation in unpaid labor since the mid-1900s (e.g., men’s increase in parental leave and increased time spent on domestic tasks in heterosexual households). A graph illustrated the percentage of parental leave taken by men and women since from 1974 to now (see Figure A1 in Appendix A).

#### Role Non-traditionalism

Perceived role non-traditionalism was estimated as in Study 1^4^: communal non-traditionalism included men’s participation in communal occupations and household tasks (α = 0.89), whereas agentic non-traditionalism included women’s participation in agentic occupations and household tasks (α = 0.90).

#### Gender Stereotypic Characteristics

The gender stereotypic characteristics scales used in Study 1 were abbreviated in order to avoid participant fatigue that was deemed to be of greater concern in this study, due to the presence of a text for the participants to read. The scales were first constructed to be divided along valence to create a positive and negative scale for both femininity and masculinity. However, the scale for positive masculinity showed very poor reliability; α = 0.58 after trimming of an item with low inter-item correlation. Considering that negative characteristics were included in previous studies on dynamic stereotypes to avoid the risk of confusing stereotype change with social desirability (Diekman and Eagly, 2000), we chose to use measures of combined positive and negative femininity/masculinity; given that regardless of valence the items should be correlated within each gender stereotype. The new, combined scales were made up of eight items for each scale (four positive and four negative items)^5^. Reliability was good for both the femininity scale (α = 0.71) and for the masculinity scale (α = 0.81). Participants responded in terms of how likely on a scale from 1 (*not at all likely*) to 7 (*very likely*) a woman/man in 1950/2018 would be to possess these characteristics.

### Results

#### Perceived Role Non-traditionalism

To test if the framing of role change influenced perceived role non-traditionalism between times, we performed a 3 (Framing of Role Change) × 2 (Target Gender) × 2 (Year) × 2 (Type of Non-traditionalism) mixed ANOVA with type of role non-traditionalism as a within-subjects factor and framing of role change, year, and target gender as between-subjects factors. Non-traditionalism increased over time, *F*(1,641) = 310.00, *p* < 0.001, $\eta_{p}^{2}$ = 0.33, but none of the other expected effects were significant (*p*’s > 0.05); indicating that participants rated agentic and communal non-traditionalism similarly independent of conditions (see Table 7 for descriptive data). As in Study 1, participants estimated higher non-traditionalism than actual distributions in all gender-typical occupations (see Table 8 for descriptive data on estimated gender distribution of occupations compared to official statistics and Table 9 for descriptive data on estimated gender division of household tasks).

**Table 7:** Study 2: Means and standard deviations by year and framing of role change for role non-traditionalism.

	Communal role non-traditionalism		Agentic role non-traditionalism	
Framing of role change	*M*	*SD*	*M*	*SD*
Men in communal roles				
1950	17.04_ a_	11.37	15.72_a_	14.60
2018	31.43_ b_	9.61	32.24_b_	11.94
Women in agentic roles				
1950	17.93_a_	13.38	16.64_a_	15.49
2018	34.36_b_	9.70	34.30_b_	12.07
Control group				
1950	17.60_a_	11.87	16.18_b_	14.47
2018	31.97_b_	9.46	31.69_b_	11.69


*Ratings of role non-traditionalism were made through estimating percentages (0–100) of women and men occupying social roles. Means with different subscripts across time points differ significantly at p < 0.05*.

**Table 8:** Study 2: Mean estimates of percentages of women working in different occupations compared to official labor statistics (Statistics Sweden, 2016).

	Estimated percentage of women in occupations	
Occupations	Participant estimates	Statistics Sweden statistics
Car mechanic	20.79	2.98
Pilot	27.72	7.19
Civil engineer	39.65	16.01
Stock broker	37.77	23.62
Police officer	40.18	40.18
Nurse	70.86	89.52
Pre-school teacher	75.81	95.66
Receptionist	71.83	80.14
Midwife	83.09	99.57
Social worker	69.27	85.61


*0 = only men in the profession, 100 = only women in the profession.*

**Table 9:** Study 2: Percentage of household tasks performed by the woman in a heterosexual household with children by year.

	Time			
	1950		2018	
Household task	*M*	*SD*	*M*	*SD*
Car repairs	11.34	17.12	28.04	16.25
Changing lightbulbs	28.09	22.07	42.81	14.58
Home repairs	21.55	19.92	34.58	14.74
Solving technology problems	17.23	19.15	33.51	15.25
Cleaning	88.13	17.56	64.81	13.14
Laundry	90.70	15.47	65.66	13.37
Cooking	86.72	17.57	60.01	11.38
Playing with children	66.50	19.15	55.58	11.49
Assisting children with homework	69.78	19.81	58.63	11.53
Caring for children’s appearance	85.73	17.43	66.68	15.06


*Ratings of performance of household tasks were made through estimating percentage of task performed by either the woman or the man in a heterosexual relationship: 0 = task only performed by man, 100 = task only performed by woman.*

#### Gender Stereotype Content

To test whether the framing of role change influenced perceived characteristics of women and men, two 3 (Framing of Role Change) × 2 (Target Gender) × 2 (Year) ANOVAs were computed.

For femininity, there was a significant main effect of target gender, *F*(1,633) = 285.89, *p* < 0.001, $\eta_{p}^{2}$ = 0.31: Women were perceived as more likely to possess feminine personality characteristics than men. There was no significant main effect of time, *F*(1,633) = 0.06, *p* = 0.81, $\eta_{p}^{2}$ < 0.01, or framing of role change, *F*(2,633) = 0.21, *p* = 0.81, $\eta_{p}^{2}$ < 0.01. A significant interaction effect between time and gender, *F*(1,633) = 13.18, *p* < 0.001, $\eta_{p}^{2}$ = 0.02, indicated that women in 2018 were perceived to have lower levels of femininity than in 1950 (*p* = 0.04), whereas the perceived change for men was not significant (*p* = 0.90). Women were still seen as more feminine than men in 2018, (*p* < 0.001), but the gender gap was smaller than for 1950, (*p* < 0.001, see Table 10 for mean values). Finally, different framings of role change did not influence perceptions of women and men differently, since the framing of role change did not interact with time and gender, *F*(2,633) = 0.76, *p* = 0.47, $\eta_{p}^{2}$ = 0.002, meaning that focusing on women’s or men’s actual change did not differentially influence perceptions of women and men over time with regards to feminine personality.

**Table 10:** Study 2: Means and standard deviations for feminine personality by framing of role change, year, and target gender.

	Men in communal roles	Women in agentic roles	Control group	Total
Target year and gender	*M (SD)*	*M (SD)*	*M (SD)*	*M (SD)*
Women				
1950	5.02_a1_ (0.71)	4.96_a1_ (0.61)	5.13_a1_ (0.67)	5.05_a1_ (0.67)
2018	4.76_a1_ (0.84)	4.79_a1_ (0.57)	4.94_a1_ (0.70)	4.84_b1_ (0.70)
Men				
1950	4.02_a2_ (0.77)	3.87_a2_ (0.60)	3.94_a2_ (0.76)	3.93_a2_ (0.71)
2018	4.09_a2_ (0.62)	4.26_a2_ (0.40)	4.02_a2_ (0.63)	4.11_a2_ (0.58)
Total	4.50 (0.85)	4.43_a2_ (0.71)	4.51_a2_ (0.87)	


*Within each target gender, means with different subscripts (a, b) differ significantly at p < 0.05 between time points. Within each time point, means with different subscripts (1, 2) differ significantly at p < 0.05 between women and men. Ratings were on a 7-point scale on which higher numbers indicate greater likelihood of possessing the characteristics*.

**FIGURE 3:**
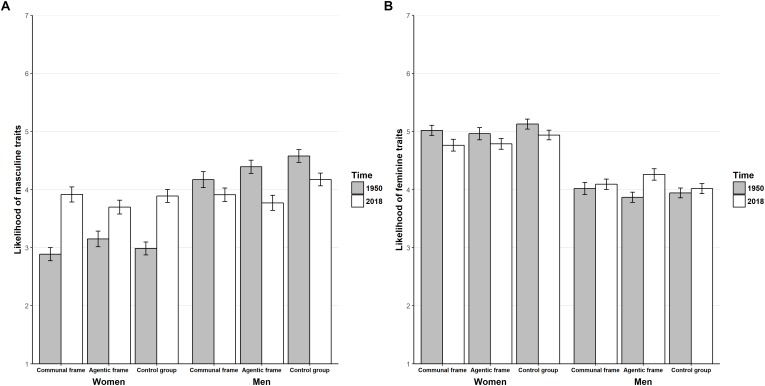
**(A)** change in masculine characteristics over time by framing of role change and target gender, **(B)** change in feminine characteristics over time by framing of role change and target gender. Error bars represent standard errors of the means.

For masculinity, there was a significant main effect of target gender *F*(1,633) = 121.98, *p* < 0.001, $\eta_{p}^{2}$ = 0.16, a significant main effect of year, *F*(1,633) = 7.04, *p* = 0.01, $\eta_{p}^{2}$ = 0.01, and a significant main effect of framing of role change *F*(2,633) = 3.61, *p* = 0.03, $\eta_{p}^{2}$ = 0.01. However, framing of role change did not significantly interact with target gender, *F*(2,633) = 1.80, *p* = 0.17, $\eta_{p}^{2}$ = 0.01, or time, *F*(2,633) = 2.49, *p* = 0.08, $\eta_{p}^{2}$ = 0.01. Instead, reading about men’s increase in communal roles decreased ratings of masculine characteristics in comparison to the control condition (*p* < 0.01), but not in comparison to reading about agentic role change (*p* = 0.27). See Table 11 for mean values and standard deviations. There was a significant interaction between time and target gender, *F*(1,633) = 78.49, *p* < 0.001, $\eta_{p}^{2}$ = 0.11. Pairwise comparisons showed that women were seen as increasing in masculine characteristics from 1950 to 2018 (*p* < 0.001), but also that men were seen as decreasing in masculine characteristics from 1950 to 2018 (*p* < 0.001), leading to the gender gap disappearing for 2018 (*p* = 0.24; in 1950, men were seen as more masculine than women). Finally, we found limited support that different framings of role change influenced perceptions of women and men differently: there was no significant omnibus interaction between framing of role change, time and gender, *F*(2,633) = 0.11, *p* = 0.90, $\eta_{p}^{2}$ < 0.001, but pairwise comparisons did show that men’s decrease in masculinity only was significant within the condition which described women’s increasing occupancy of agentic roles (*p* = 0.02). See Figure 3 for a visualization of perceived stereotype content change over time by framing of role change.

**Table 11:** Study 2: Means and standard deviations for masculine personality by framing of role change, year, and target gender.

	Men in Communal roles	Women in agentic roles	Control group	Total
Target year and gender	*M (SD)*	*M (SD)*	*M (SD)*	*M (SD)*
Women				
1950	2.89_a1_ (0.77)	3.15_a1_ (0.88)	2.99_a1_ (1.04)	2.99_a1_ (0.91)
2018	3.82_b1_ (0.90)	3.70_a1_ (0.78)	3.89_b1_ (0.86)	3.81_b1_ (0.85)
Men				
1950	4.17_a2_ (0.94)	4.39_a2_ (0.92)	4.58_a2_ (0.88)	4.41_a2_ (0.92)
2018	3.91_a1_ (0.86)	3.77_b1_ (0.66)	4.18_a1_ (0.81)	3.97_b1_ (0.80)
Total	3.64 (0.99)	3.81 (0.93)	3.92 (1.07)	


*Within each target gender, means with different subscripts (a, b) differ significantly at p < 0.05 between time points. Within each time point, means with different subscripts (1, 2) differ significantly at p < 0.05 between women and men. Ratings were on a 7-point scale on which higher numbers indicate greater likelihood of possessing the characteristics*.

#### Correspondence Between Roles and Gender Stereotypes

Similar mediation models and analysis method as in Study 1 were used to test whether role distribution influenced gender stereotype content, with the addition of adding framing of role change as a covariate. Framing of role change was dummy coded, with control group as reference, and added as a covariate rather than as a possible moderator since it had not displayed any interaction effects with variables in previous analyses.

For masculinity, the index of moderated mediation showed an indirect effect of time through agentic role non-traditionalism moderated by target gender, indicating that the indirect effect of time through agentic non-traditionalism differed based on target gender. The effect of time was mediated for perception of women targets, *b* = 0.28, *SE* = 0.09, *LLCI* = 0.14, *ULCI* = 0.48, but not for perception of men targets, *b* = -0.10, *SE* = 0.10, *LLCI* = -0.31, *ULCI* = 0.07. There was also a significant direct effect of time on masculinity, which was moderated by target gender: For women targets the effect of time was positive, *b* = 0.52, *SE* = 0.11, *LLCI* = 0.30, *ULCI* = 0.73, and for men targets the effect of time was negative, *b* = -0.34, *SE* = 0.11, *LLCI* = -0.56, *ULCI* = -0.12. Women’s increase in masculinity partially was an effect of increased occupancy of agentically demanding roles, whereas men’s decrease in masculinity was not a result of decreased occupancy of agentically demanding roles. There was also a general increase in these characteristics over time for women which was unrelated to social role occupancy. See Table 12 for path coefficients and index of moderated mediation.

**Table 12:** Study 2: Unstandardized regression coefficients (standard errors in parentheses) with confidence intervals for estimating the indirect conditional effect of time on masculine personality through agentic role non-traditionalism, moderated by target gender.

	Agentic role non-traditionalism		Masculine personality	
Predictor	*b*	95% *CI*	*b*	95% *CI*
Time	16.49^∗∗∗^ (1.06)	14.41, 18.57	0.52^∗∗∗^ (0.11)	0.30, 0.73
Women in agentic roles	1.54 (1.28)	–0.98, 4.05	–0.14 (0.08)	–0.30, 0.02
Men in communal roles	0.04 (1.28)	–2.48, 2.55	–0.21^∗^ (0.08)	–0.37, –0.05
Agentic role non-traditionalism			0.02^∗∗∗^ (0.003)	0.01, 0.02
Gender			1.78^∗∗∗^ (0.13)	1.53, 2.02
Time × Gender			–0.85^∗∗∗^ (0.16)	–1.16, –0.54
Agentic role Non-traditionalism × Gender			–0.02^∗∗∗^ (0.01)	–0.03, –0.01
				
Constant	15.68^∗∗∗^ (1.01) *R*^2^ = 0.28	13.70, 17.66	2.83^∗∗∗^ (0.10) *R*^2^ = 0.30	2.64, 3.02
	*F*(3,640) = 81.17, *p* < 0.001		*F*(7,636) = 38.26, *p* < 0.001	
Index of moderated mediation			Index = –0.38, 95% CI = –0.68, –0.15	


*^∗^*p* < 0.05, ^∗∗^*p* < 0.01, ^∗∗∗^*p* < 0.001.*

In contrast to Study 1, there was also an indirect effect of time on masculinity through communal non-traditionalism, moderated by target gender. The effect of time was mediated for perception of both women, *b* = 0.23, *SE* = 0.10, *LLCI* = 0.09, *ULCI* = 0.46, and men targets, *b* = -0.20, *SE* = 0.10, *LLCI* = -0.43, *ULCI* = -0.03. There was also a significant direct effect of time on masculinity which was moderated by target gender, for women targets the effect of time was positive, *b* = 0.57, *SE* = 0.12, *LLCI* = 0.34, *ULCI* = 0.80, and for men targets the effect of time was negative, *b* = -0.23, *SE* = 0.12, *LLCI* = -0.46, *ULCI* = -0.01. This analysis shows that women’s increase in masculinity partially was an effect of decreased occupancy in communally demanding roles, whereas men’s decrease in masculinity partially was a result of increased occupancy in communally demanding roles. There was also a general increase in these characteristics over time for women as well as a decrease for men which was unrelated to social role occupancy. See Table 13 for path coefficients and index of moderated mediation.

**Table 13:** Study 2: Unstandardized regression coefficients (standard errors in parentheses) with confidence intervals for estimating the indirect conditional effect of time on masculine personality through communal non-traditionalism, moderated by target gender.

	Communal role non-traditionalism		Masculine personality	
Predictor	*b*	95% *CI*	*b*	95% *CI*
Time	15.00^∗∗∗^ (0.87)	13.30, 16.69	0.57^∗∗∗^ (0.12)	0.34, 0.80
Women in agentic roles	1.41 (1.05)	–0.64, 3.47	–0.15 (0.08)	–0.31, 0.01
Men in communal roles	–0.56 (1.05)	–2.62, 1.49	–0.21^∗^ (0.08)	–0.37, –0.05
Communal role non-traditionalism			0.02^∗∗∗^ (0.004)	0.01, 0.02
Gender			1.92^∗∗∗^ (0.14)	1.63, 2.20
Time × Gender			–0.80^∗∗∗^ (0.16)	–1.12, –0.48
Communal role non-traditionalism × Gender			–0.03^∗∗∗^ (0.01)	–0.04, –0.02
				
Constant	17.28^∗∗∗^ (0.82) *R^2^* = 0.32	15.66, 18.90	2.84^∗∗∗^ (0.11) *R*^2^ = 0.29	2.61, 3.05
	*F*(3,640) = 101.15, *p* < 0.001		*F*(7,636) = 37.36, *p* < 0.001	
Index of moderated mediation			Index = –0.43, 95% CI = –0.77, –0.19	


*^∗^p < 0.05, ^∗∗^p < 0.01, ^∗∗∗^p < 0.001.*

Taken together, these analyses show that women are seen as increasing in masculinity both when they are increasingly found in agentically demanding roles, and when they decrease their participation in communally demanding roles. On the other hand, perception of men’s masculinity is not affected by their participation in agentically demanding roles, but decreases when men are seen as increasingly occupying communal roles.

In contrast to the expectations, we found neither a mediation by communal or agentic non-traditionalism for femininity, nor an indirect effect conditional on gender (see Appendix D, Tables D4, D5 for analysis details). Therefore, as in Study 1, time was not found to have an indirect effect on femininity through communal non-traditionalism conditional on gender.

### Discussion

Study 2 presented actual changes in gendered division of labor from the past to the present alongside a control condition to test if participants’ estimates of role non-traditionalism and gender stereotype content were affected by a Swedish equality bias. Contrary to expectations, presenting participants with information about actual change regarding social role occupancy did not diminish overestimations of role non-traditionalism compared to the control condition. Instead, participants in Study 2, including the control condition, overestimated the prevalence of women and men in non-traditional roles.

Furthermore, framing of role change had only a limited effect on gender stereotype content. The framing which described men’s increase in communal roles did not affect the perceived femininity of men – instead, such a framing decreased overall perceptions of masculinity. However, men’s decrease in masculinity from the past to the present was only significant in the condition which described women’s increase occupancy of agentic roles; indicating that framing women’s increased occupancy of agentic roles lead to decreased perceptions of masculinity in men of the present.

Regardless of how we framed role change, there was support for specifically the stereotype of women being seen as dynamic. Women were perceived as both more masculine and less feminine today compared to the past. Mediation analyses indicated that the perceived increase for women in masculinity was partially explained by their increased participation in agentically demanding roles, along with a decrease in communal role occupation. However, women’s perceived decrease in femininity from the past to the present was not mediated by social role occupancy.

The male stereotype was not subject to an increase in gender-atypical characteristics as the female stereotype was, but men were seen as having less masculine characteristics today than in the past. Mediation analyses, indicated that men’s perceived decrease in masculinity was partly qualified by an increased degree of participation in communally demanding roles. This indicates that when men engage in communal roles this may in fact contribute to a loss of masculinity rather than a gain in femininity. Such results are in line with the ideas of the precarious manhood (Vandello et al., 2008), because there may be differences in the malleability of masculinity and femininity, where masculinity is easier to gain and lose, whereas femininity for men is more difficult to attain but can be lost by women through some mechanism other than social role occupancy.

## General Discussion

Social role theory explains the origins of gender stereotypes by observed division of labor (Eagly, 1987). Even though people often consider gender stereotypes as relatively stable characteristics, previous research shows that traits associated with women and men are dynamic and subject to change (Diekman and Eagly, 2000; Diekman et al., 2005; Wilde and Diekman, 2005). Such dynamics are expected from social role theory, as changes in the division of labor should be accompanied by corresponding changes in perceived traits of women and men. Changes in division of labor could occur due to several factors. For example, it might be due to economic factors or due to ideological factors, such as national gender equality goals. One example of the latter is campaigns about paternity leave in Sweden which are grounded in an active political striving to increase men’s participation in child-care.

In line with such changes, past research (Diekman et al., 2005; Wilde and Diekman, 2005; Bosak et al., 2017) has consistently shown that the female stereotype is seen as more agentic over time, whereas results about perceived change in the male stereotype are mixed. This may be due to contextual variations regarding to what extent men have entered communal roles. In order to test the assumption that men’s engagement in communal roles affects the male stereotype, the present research tested whether the male stereotype has changed in line with predictions from social role theory in one of the world’s most egalitarian countries – Sweden. Based on several indicators of high gender equality and one of the most beneficial parental leave systems for fathers, we assumed that the male stereotype should include more communion in a Swedish sample as compared to most other countries.

In two studies, we tested if Swedish participants believed that characteristics of women and men changed from the present to the future (Study 1), and from the past to the present (Studies 1 and 2). The results of both studies showed that the content of the female stereotype increased in masculinity. Furthermore, the female and male stereotype converged on masculinity for evaluation of a target in the present. Thus, at the present time, Swedish women and men are seen as equally masculine. This result supports the notion of Sweden as being one of the most egalitarian countries in the world. Moreover, the result for stereotypes in the present somewhat align with how masculinity is estimated in women and men in the future from similar studies in other countries (see for example Garcia-Retamero et al., 2011; Bosak et al., 2017). We did not see any future change on both of the personality dimensions, which could be explained by this convergence occurring for the present. If women and men are currently perceived as equally masculine, and if this convergence reflects the notion of being gender equal, there is no need for further change in the future. Hence, the lack of future change is in line with the idea and discourse of Sweden as already having arrived at an end point of gender equality; in other words, where equality has already been reached. However, given that large gender differences still exist in Sweden’s gender segregated labor market, e.g., women take longer parental leave than men, gender equality is still to be reached. But, as indicated by our results, the movement toward gender equality might proceed at a much slower pace in the future. If people believe that equality has already been reached and perceive women and men as being alike, the existence of gender segregation might be attributed to individual preferences regarding interest in specific occupations rather than to gender stereotypes: which under the current neoliberal framework might be seen as less important to change. Future research should therefore study the origins and consequences of dissonance between actual and perceived gender segregation. For example, the media often strive to present counter-stereotypical representatives. Although these aims are well intended and probably serve as important role models, such strategies might also backlash into false understandings of the actual gender distribution of the labor market.

At the same time, we found no increase in the perceived communality of men. Because Sweden is highly egalitarian, and because Swedish fathers take more parental leave than any other fathers in the world, we expected that there would be a perceived increase in feminine traits among men from past to present time. This expectation was not confirmed in any of the studies, even though participants perceived increased numbers of men in communal roles in both studies. In Study 1, participants overestimated the amount of men in communal roles and in Study 2, they were presented with statistical facts showing the increase in fathers’ parental leave from 1974 (the introduction of parental leave rather than maternal leave) to 2017. These facts showed a trend over time which clearly indicated that men have become increasingly engaged in the communally demanding task of child-rearing. Still, there was no corresponding increase in perceived femininity of men. Even though participants did not perceive a change in men’s feminine traits, the Swedish male stereotype is quite balanced on agency and communion, as revealed when more closely examining the means for men’s femininity and masculinity scores.

Several explanations for the difference in malleability of traits associated with masculinity or femininity are possible, and should be more closely studied in future research. One supposition is that the perceived traits associated with masculinity are more malleable than traits associated with femininity. Past studies have shown that social roles were more strongly correlated with agentic than communal behavior (Moskowitz et al., 1994). Masculine traits may be easier to gain because they are viewed as performative rather than essential. Hence, as women engage in agentic roles, they are perceived to gain agentic traits. Feminine traits, especially those that relate to nurturing and care-taking, on the other hand, might be seen as more essential to the category ‘woman’ and more difficult to gain through role occupation (McPherson et al., 2018). Following this, even if men engage in communal roles, they may not be perceived to gain communal traits. In support of this idea, the results from Study 2 showed that when men entered communal roles, they were not perceived as having gained communal/feminine traits. Presenting participants with facts about men’s increased participation in communal roles did not affect ratings of femininity, and perception of a higher degree of men in communal roles instead mediated the decrease in men’s perceived masculinity over time. Some scholars argue that biology might be one cue that fosters essentialism, and if women’s caregiving is perceived as more related to biology than men’s (McPherson et al., 2018) this could be one factor explaining why men did not increase in communal traits. Yet another reason might be that the number of men in caregiving roles are still too few to cause a change in perceived communality. Finally, mothers and fathers may enact caregiving in a range of different ways – that is, simply because a man is home for parental leave does not mean that he is engaging in caregiving behaviors in the same way that women do. In comparison to roles in the labor market, specific family roles might be easier to adjust to broad gender stereotypes.

Moreover, in Study 1, the female stereotype had higher values on positive traits associated with femininity than the male stereotype across all three time points, indicating a stability in gender differences. This could be explained by the fact that even though Swedish fathers’ take comparatively more parental leave than in other countries, Swedish mothers still take the bulk of the parental leave, and they also work in more communal sectors on the labor market. Furthermore, men have not entered communal occupations as shown by official statistics (Statistics Sweden, 2016) meaning that the Swedish labor market is still gender segregated along the lines of communion/agency. Hence, even though Sweden is highly ranked on national indices of gender equality, it is actually not gender equal, even though many of its citizens seem to believe that. The strong overestimation of gender equality in occupations supports this interpretation.

A limitation of these studies is that we have not controlled for participants’ awareness of the current gender equality situation in Sweden. In Study 1, we found strong overestimations of the extent to which women and men have entered into non-stereotypical occupations. In Study 2, we controlled for participants’ knowledge of actual changes in division of labor, however, participants still underestimated the degree of gender segregation present in the Swedish labor market. Future studies should more explicitly test whether Swedish people think that equality has been reached and whether such beliefs also influence perceptions of division of labor and gender stereotype content.

Another possible explanation of the convergence between women and men on three of the four personality dimensions might be that traits associated with femininity and masculinity differ in Sweden compared to other nations in which these stereotype dimension scales have been tested. Future research should investigate if this decrease in stereotypicality of classically gendered traits has led to a decrease in gender stereotyping, or if other traits than those used in previous research have become gendered, thus contributing to updated knowledge regarding gender differences in stereotype content.

## Conclusion

In support of social role theory, we directly showed that the perceived change in women’s agentic traits was specifically associated with a perceived change in the roles occupied by women. However, men were not perceived to change as a result of changing roles. Instead, when men were seen in non-traditional roles, their communal characteristics did not increase. Thus, seeing men taking their children to pre-school as described in the first quote is so far not enough to also perceive men as communal.

## Ethics Statement

These studies were carried out in accordance with the national guidelines on ethical research established by the Swedish Research Council retrievable at https://publikationer.vr.se/en/product/good-research-practice/. All participants gave their informed consent before participating in the survey.

## Author Contributions

MGS conceptualized the idea and wrote the manuscript. AK collected data, performed statistical analyses, and wrote the result section. All authors participated in the planning of the studies, interpretation and discussion of results, and in writing the manuscript.

## Conflict of Interest Statement

The authors declare that the research was conducted in the absence of any commercial or financial relationships that could be construed as a potential conflict of interest.

## Footnotes

1 Four professions with an equal gender distribution were added as filler items: physician, retail salesperson, journalist, and university teacher.

2 Participants rated the characteristics regarding both how likely the target was to possess them (descriptive beliefs) and how beneficial/harmful the characteristics would be for the target to possess (prescriptive beliefs) (Diekman and Goodfriend, 2006). Results for the two were aligned and therefore only results for descriptive stereotype content is presented, results for prescriptive analysis can be found in Appendix D.

3 The following items were changed: The item “aggressive” was moved to the masculine negative personality scale due to higher inter-item correlation, *r*_positive_ =0.39 and *r*_negative_ =0.73. “Unprincipled” was removed from the masculine scale and “spineless” and “subordinates self to others” was removed from the female scale to increase internal reliability.

4 Midwife and social welfare secretary were added to female-dominated professions, and taking care of sick children and paying bills was removed from domestic tasks because they were not associated strongly with either gender.

5 For scales see Appendix A.
