# Erratum: The (Not So) Changing Man: Dynamic Gender Stereotypes in Sweden

**DOI:** 10.3389/fpsyg.2019.00892

**Published:** 2019-04-04

**Authors:** Frontiers Production Office

**Affiliations:** Frontiers Media SA, Lausanne, Switzerland

**Keywords:** social role theory, gender stereotypes, femininity, masculinity, agency, communion, division of labor

Due to a typesetting error, the first paragraph and heading of the section “Perceived Role Non-Traditionalism” was incorrectly placed in the section “Sweden and Gender Equality”, it belongs in the section “Results” after the second paragraph. The publisher apologizes for this mistake.

The original version of this article has been updated.

