# Supplementary material for: The (Not So) Changing Man: Dynamic Gender Stereotypes in Sweden
Source: Front Psychol. 2019 Jan 30;10:37. doi: 10.3389/fpsyg.2019.00037 (PMC6363713; doi:10.3389/fpsyg.2019.00037)
Supplement: Supplementary file 1 [file Table_1.DOCX]

**Appendix A: Supplementary material**

**Table A1.** Items in gender stereotype content dimensions, in original English and translated into Swedish with Cronbach’s alpha values for Study 1 and 2.

|  | Communion | |  | Agency | |
| --- | --- | --- | --- | --- | --- |
| Dimension | English | Swedish |  | English | Swedish |
| Positive | Affectionate^d^ | Omsorgsfull^d^ |  | Competitive | Tävlingsinriktad |
|  | Sympathetic | Engagerad |  | Daring | Vågad |
|  | Gentle | Mild |  | Adventurous_d_ | Äventyrlig^d^ |
|  | Sensitive | Känslig |  | Aggressive^cd^ | Aggressiv^cd^ |
|  | Supportive | Stöttande |  | Courageous^d^ | Modig^d^ |
|  | Kind^d^ | Vänlig^d^ |  | Dominant^d^ | Auktoritär^d^ |
|  | Nurturing^d^ | Omhändertagande^d^ |  | Unexcitable^a^ | Behärskad^a^ |
|  | Warm^d^ | Varm^d^ |  | Stands up under pressure^de^ | Strestålig^de^ |
|  |  | α_1_ = .89 |  |  | α_1_ = .76 |
| Negative | Spineless^b^ | Ryggradslös^b^ |  | Egotistical | Självisk |
|  | Gullible | Godtrogen |  | Hostile^d^ | Stridslysten^d^ |
|  | Servile^a^ | Tjänstvillig^a^ |  | Cynical | Cynisk |
|  | Subordinates self to others^b^ | Underordnar sig andra^b^ |  | Arrogant | Arrogant |
|  | Whiny^de^ | Gnällig^de^ |  | Boastful^d^ | Skrytsam^d^ |
|  | Complaining^d^ | Missnöjd^d^ |  | Greedy | Girig |
|  | Nagging^d^ | Tjatig^d^ |  | Dictatorial^d^ | Diktatorisk^d^ |
|  | Fussy^d^ | Petig^d^ |  | Unprincipled^b^ | Principlös^b^ |
|  |  | α_1_ = .74, α_2_ = .71 |  |  | α_1_ = .91, α_2_ = .81 |
| Cognitive | Imaginative | Fantasirik |  | Good with numbers | Bra med siffror |
|  | Intuitive | Intuitiv |  | Analytical | Analytisk |
|  | Artistic | Konstnärlig |  | Good at problem solving | Bra på problemlösning |
|  | Creative | Kreativ |  | Quantitatively skilled | Bra på beräkningar |
|  | Expressive | Uttrycksfull |  | Good at reasoning | Bra på logiska resonemang |
|  | Tasteful | Har god smak |  | Mathematical | Matematisk |
|  |  | α_1_ = .83 |  |  | α_1_ = .91 |
| Physical | Cute | Söt |  | Rugged | Robust byggd |
|  | Gorgeous | Snygg |  | Muscular | Muskulös |
|  | Beautiful | Vacker |  | Physically strong | Fysiskt stark |
|  | Pretty | Fin |  | Burly | Kraftigt byggd |
|  | Petite^b^ | Nätt^b^ |  | Physically vigorous | Energisk |
|  | Sexy | Sexig |  | Brawny | Atletisk |
|  |  | α_1_ = .87 |  |  | α_1_ = .81 |

*Note.* Unmarked items are final scales used in Study 1: a) Items were removed following a pilot study, b) Items were removed due to low internal reliability, c) Moved to subscale ‘masculine negative personality’ due to higher correlation with negative items than positive, d) Items used in study 2, e) Items removed from Study 2 due to low internal reliability


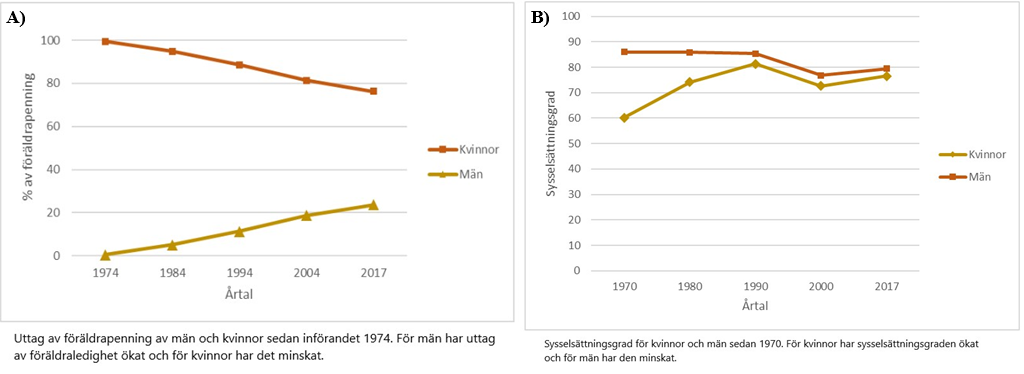


**Figure A1.** Graphs accompanying the framing of role change texts used in Study 2. **A)** Change in men’s and women’s parental leave from 1974 to 2017, **B)** Change in women’s and men’s labor market participation from 1970 to 2017.
