# Supplementary material for: The (Not So) Changing Man: Dynamic Gender Stereotypes in Sweden
Source: Front Psychol. 2019 Jan 30;10:37. doi: 10.3389/fpsyg.2019.00037 (PMC6363713; doi:10.3389/fpsyg.2019.00037)
Supplement: Supplementary file 2 [file Table_2.DOCX]

**Appendix B: Analysis details of the effect of year, target gender, and year x gender for gender stereotype content**

**Table B1.** Analysis of the effect of year, target gender, and year x gender for descriptive gender stereotype content

|  | Stereotype Dimension | | | | | | | | | | | | | | | | | | | | | | |
| --- | --- | --- | --- | --- | --- | --- | --- | --- | --- | --- | --- | --- | --- | --- | --- | --- | --- | --- | --- | --- | --- | --- | --- |
|  | Femininity | | | | | | | | | | |  | Masculinity | | | | | | | | | | |
|  | Personality positive | |  | Personality negative | |  | Cognitive | |  | Physical | |  | Personality positive | |  | Personality negative | |  | Cognitive | |  | Physical | |
|  | *F* | η^2^_p_ |  | *F* | η^2^_p_ |  | *F* | η^2^_p_ |  | *F* | η^2^_p_ |  | *F* | η^2^_p_ |  | *F* | η^2^_p_ |  | *F* | η^2^_p_ |  | *F* | η^2^_p_ |
| Target year (2) | 1.39 | 0.01 |  | 5.97** | 0.04 |  | 10.13*** | 0.06 |  | 2.13 | 0.13 |  | 15.53*** | 0.09 |  | 24.25*** | 0.13 |  | 9.28*** | 0.06 |  | 0.90 | 0.01 |
| Target gender (1) | 71.55*** | 0.18 |  | 0.39 | 0.001 |  | 24.39*** | 0.07 |  | 45.39*** | 0.13 |  | 6.30* | 0.02 |  | 31.31*** | 0.09 |  | 0.19 | 0.001 |  | 9.11** | 0.03 |
| Year x Gender (2) | 3.71* | 0.02 |  | 0.84 | 0.01 |  | 0.41 | 0.003 |  | 1.07 | 0.01 |  | 4.41* | 0.03 |  | 2.90^†^ | 0.02 |  | 0.06 | <0.001 |  | 0.43 | 0.003 |
| *R^2^* | .21 | |  | .043 | |  | .12 | |  | .14 | |  | .13 | |  | .22 | |  | .057 | |  | .036 | |

*Note.* ^†^*p* <.06, **p*<.05, ***p*<.01, ****p*<.001. *df1* is presented by its corresponding factor, and *df2* = 317 for all dimensions.

**Table B2.** Mean and standard deviation by target gender for cognitive and physical descriptive stereotype dimensions

|  | Stereotype dimension | | | | | | |
| --- | --- | --- | --- | --- | --- | --- | --- |
|  | Masculinity | | |  | Femininity | | |
|  | Cognitive |  | Physical |  | Cognitive |  | Physical |
|  | *M (SD)* |  | *M (SD)* |  | *M (SD)* |  | *M (SD)* |
| Woman |  |  |  |  |  |  |  |
| 1950 | 3.82_a1_ (1.11) |  | 3.60_a1_ (1.14) |  | 4.09_a1_ (0.98) |  | 4.35_a1_ (1.11) |
| 2017 | 4.45_b1_ (1.04) |  | 3.87_a1_ (0.97) |  | 4.54_b1_ (0.85) |  | 4.51_a1_ (0.91) |
| 2090 | 4.24_b1_ (0.89) |  | 3.70_a1_ (0.69) |  | 4.35_b1_ (0.72) |  | 4.25_a1_ (0.96) |
| **Total** | 4.16 (1.05) |  | 3.72 (0.95) |  | 4.32 (0.87) |  | 4.36 (1.00) |
| Man |  |  |  |  |  |  |  |
| 1950 | 3.92_a1_ (1.09) |  | 4.05_a2_ (0.93) |  | 3.50_a2_ (1.06) |  | 3.42_a2_ (1.05) |
| 2017 | 4.51_b1_ (0.95) |  | 4.09_a1_ (0.90) |  | 4.10_b2_ (0.75) |  | 3.81_b2_ (0.87) |
| 2090 | 4.24_b1_ (1.05) |  | 3.98_a1_ (0.92) |  | 3.96_b2_ (0.76) |  | 3.71_b2_ (0.84) |
| **Total** | 4.22 (1.05) |  | 4.04 (0.91) |  | 3.86 (0.89) |  | 3.65 (0.93) |

*Note.* Within each target gender, means with different column subscripts (a,b) differ significantly at *p* < .05 between time points. Within each time point, means with different column subscripts (1,2) differ significantly at *p* < .05 between women and men.
