# Supplementary material for: The (Not So) Changing Man: Dynamic Gender Stereotypes in Sweden
Source: Front Psychol. 2019 Jan 30;10:37. doi: 10.3389/fpsyg.2019.00037 (PMC6363713; doi:10.3389/fpsyg.2019.00037)
Supplement: Supplementary file 3 [file Table_3.DOCX]

**Appendix C: Moderated mediation analyses**

**Table C1.** Study 1. Unstandardized regression coefficients (standard errors in parentheses) with confidence intervals for estimating the indirect conditional effect of time on masculine stereotype dimensions through communal nontraditionalism, moderated by target gender.

|  | Communal role nontraditionalism | |  | Masculine personality positive | |  | Masculine personality negative | |  | Masculine cognitive | |  | Masculine physical | |
| --- | --- | --- | --- | --- | --- | --- | --- | --- | --- | --- | --- | --- | --- | --- |
| Predictors | *b* | 95% CI |  | *b* | 95% CI |  | *b* | 95% CI |  | *b* | 95% CI |  | *b* | 95% CI |
| Time | 10.37*** (0.76) | 8.60, 11.87 |  | 0.30** (0.11) | 0.08, 0.51 |  | 0.31* (0.13) | 0.05, 0.57 |  | 0.12 (0.13) | -0.15, 0.38 |  | 0.06 (0.12) | -0.18, 0.29 |
| Communal role nontraditionalism |  |  |  | 0.01 (0.01) | -0.01, 0.02 |  | 0.02* (0.01) | 0.001, 0.03 |  | 0.01 (0.01) | -0.01, 0.02 |  | 0.0001 (0.01) | -0.02, 0.02 |
| Gender |  |  |  | 0.20 (0.25) | -0.30, 0.70 |  | 0.94** (0.31) | 0.34, 1.54 |  | -0.05 (0.31) | -0.07, 0.56 |  | 0.27 (0.28) | -.28, 0.82 |
| Time x Gender |  |  |  | -0.26 (0.15) | -0.55, 0.03 |  | -0.04 (0.18) | -0.39, 0.31 |  | -0.07 (0.18) | -0.42, 0.28 |  | -0.11 (0.16) | -0.43, 0.21 |
| Communal role nontraditionalism x Gender |  |  |  | 0.01 (0.01) | -0.02, 0.02 |  | -0.01 (0.01) | -0.03, 0.01 |  | 0.003 (0.01) | -0.02, 0.02 |  | 0.002 (0.01) | -0.02, 0.02 |
| Constant | 27.04*** (0.63) | 25.80, 28.28 |  | 3.63*** (0.19) | 3.27, 4.00 |  | 2.69*** (0.23) | 2.25, 3.13 |  | 3.98*** (0.23) | 3.53, 4.43 |  | 3.71*** (0.21) | 3.31, 4.12 |
|  | *R*^2^=.37 | |  | *R*^2^=.09 | |  | *R*^2^=.19 | |  | *R*^2^=.029 | |  | *R*^2^=.030 | |
|  | *F*(1,319)=184.09, *p* < .001 | |  | *F*(5,315)=6.53, *p* < .001 | |  | *F*(5,315)=14.57, *p* < .001 | |  | *F*(5,315)=1.89, *p* = .096 | |  | *F*(5,315)=1.97, *p* = .082 | |
| Index of moderated mediation |  | |  | Index = 0.009  95% CI = -0.22, 0.21 | |  | Index = -0.13  95% CI = -0.36, 0.08 | |  | Index = 0.027  95% CI = -0.18, 0.25 | |  | Index = 0.02  95% CI = -0.20, 0.22 | |

*Note.*  **p*<.05, ***p*<.01, ****p*<.001

**Table C2.** Study 1. Unstandardized regression coefficients (standard errors in parentheses) with confidence intervals for estimating the indirect conditional effect of time on feminine stereotype dimensions through communal nontraditionalism, moderated by target gender.

|  | Communal role nontraditionalism | |  | Feminine personality positive | |  | Feminine personality negative | |  | Feminine cognitive | |  | Feminine physical | |
| --- | --- | --- | --- | --- | --- | --- | --- | --- | --- | --- | --- | --- | --- | --- |
| Predictors | *b* | 95% CI |  | *b* | 95% CI |  | *b* | 95% CI |  | *b* | 95% CI |  | *b* | 95% CI |
| Time | 10.37*** (0.76) | 8.86, 11.87 |  | -0.23* (0.11) | -0.45, -0.004 |  | 0.09 (0.12) | -0.15, 0.32 |  | -0.01 (0.11) | -0.23, 0.21 |  | -0.05 (0.12) | -0.29, 0.20 |
| Communal role nontraditionalism |  |  |  | -0.002 (0.01) | -0.02, 0.01 |  | 0.01 (0.01) | -0.01, 0.02 |  | 0.01 (0.001) | -0.001, 0.03 |  | -0.001 (0.01) | -0.02, 0.01 |
| Gender |  |  |  | -1.19*** (0.26) | -1.70, -0.68 |  | -0.18 (0.28) | -0.73, 0.36 |  | -0.59* (0.26) | -1.09, -0.90 |  | -1.11*** (0.29) | -1.67, -0.55 |
| Time x Gender |  |  |  | 0.17 (0.15) | -0.55, 0.04 |  | 0.03 (0.18) | -0.29, 0.35 |  | 0.08 (0.15) | -0.21, 0.37 |  | 0.06 (0.17) | -0.26, 0.39 |
| Communal role nontraditionalism x Gender |  |  |  | 0.01 (0.01) | -0.01, 0.03 |  | 0.004 (0.01) | -0.02, 0.02 |  | 0.003 (0.01) | -0.02, 0.02 |  | 0.01 (0.01) | -0.01, 0.03 |
| Constant | 27.04*** (0.63) | 25.80, 28.29 |  | 4.78*** (0.19) | 4.41, 5.16 |  | 3.64*** (0.20) | 2.04, 3.18 |  | 4.00*** (0.19) | 3.39, 4.55 |  | 4.41*** (0.21) | 3.99, 4.82 |
|  | *R*^2^=.37 | |  | *R*^2^=.21 | |  | *R*^2^=.029 | |  | *R*^2^=.12 | |  | *R*^2^=.15 | |
|  | *F*(1,319)=184.09, *p* < .001 | |  | *F*(5,315)=17.20, *p* < .001 | |  | *F*(5,315)=1.88, *p* = .098 | |  | *F*(5,315)=8.69, *p* <.001 | |  | *F*(5,315)=10.40, *p* < .001 | |
| Index of moderated mediation |  | |  | Index = 0.13  95% CI = -0.06, 0.36 | |  | Index = 0.04  95% CI = -0.18, 0.24 | |  | Index = 0.03  95% CI = -0.16, 0.25 | |  | Index = 0.14  95% CI = -0.11, 0.39 | |

*Note.*  **p*<.05, ***p*<.01, ****p*<.001

**Table C3.** Study 1. Unstandardized regression coefficients (standard errors in parentheses) with confidence intervals for estimating the indirect conditional effect of time on feminine stereotype dimensions through agentic nontraditionalism, moderated by target gender.

|  | Agentic role nontraditionalism | |  | Feminine personality positive | |  | Feminine personality negative | |  | Feminine cognitive | |  | Feminine physical | |
| --- | --- | --- | --- | --- | --- | --- | --- | --- | --- | --- | --- | --- | --- | --- |
| Predictors | *b* | 95% CI |  | *b* | 95% CI |  | *b* | 95% CI |  | *b* | 95% CI |  | *b* | 95% CI |
| Time | 8.69*** (0.83) | 7.05, 10.32 |  | -0.23* (0.10) | -0.42, -0.03 |  | 0.08 (0.11) | -0.13, 0.29 |  | -0.03 (0.10) | -0.22, 0.16 |  | -0.10 (0.11) | -0.31, 0.12 |
| Agentic role nontraditionalism |  |  |  | .001 (0.01) | -0.01, 0.01 |  | 0.01 (0.01) | -0.01, 0.02 |  | 0.02** (0.01) | 0.01, 0.03 |  | 0.01 (0.01) | -0.01, 0.02 |
| Gender |  |  |  | -0.99*** (0.24) | -1.47, -0.52 |  | 0.01 (0.26) | -0.49, 0.52 |  | -0.29 (0.24) | -0.76, 0.17 |  | -0.80** (0.27) | -1.33, -0.28 |
| Time x Gender |  |  |  | 0.23 (0.14) | -0.05, 0.50 |  | 0.11 (0.15) | -0.18, 0.41 |  | 0.18 (0.14) | -0.09, 0.45 |  | 0.16 (0.15) | -0.14, 0.46 |
| Agentic role nontraditionalism x Gender |  |  |  | 0.01 (0.01) | -0.01, 0.02 |  | -0.004 (0.01) | -0.02, 0.01 |  | -0.01 (0.01) | -0.03, 0.01 |  | 0.002 (0.01) | -0.02, 0.02 |
| Constant | 26.45*** (0.69) | 25.10, 27.80 |  | 4.70*** (0.18) | 4.36, 5.04 |  | 3.64*** (0.19) | 3.28, 4.01 |  | 3.86*** (0.17) | 3.53, 4.20 |  | 4.22*** (0.19) | 3.85, 4.60 |
|  | *R*^2^ = .26 | |  | *R*^2^ = .20 | |  | *R*^2^ = .02 | |  | *R*^2^ = .13 | |  | *R*^2^ = .13 | |
|  | *F*(1,314) = 109.38, *p* < .001 | |  | *F*(5,310) = 15.62, *p* < .001 | |  | *F*(5,310) = 1.55, *p* = .17 | |  | *F*(5,310) = 9.32, *p* <.001 | |  | *F*(5,310) = 9.60, *p* < .001 | |
| Index of moderated mediation |  | |  | Index = 0.05  95% CI = -0.13, 0.22 | |  | Index = -0.03  95% CI = -0.21, 0.12 | |  | Index = -0.08  95% CI = -0.26, 0.08 | |  | Index = 0.02  95% CI = -0.17, 0.19 | |

*Note.*  **p*<.05, ***p*<.01, ****p*<.001

**Table C4.** Study 1. Unstandardized regression coefficients (standard errors in parentheses) with confidence intervals for estimating the indirect conditional effect of time on masculine cognitive and physical stereotype dimensions through agentic nontraditionalism, moderated by target gender.

|  | Agentic role nontraditionalism | |  | Masculine cognitive | |  | Masculine physical | |
| --- | --- | --- | --- | --- | --- | --- | --- | --- |
| Predictors | *b* | 95% CI |  | *b* | 95% CI |  | *b* | 95% CI |
| Time | 8.69*** (0.83) | 7.05, 10.32 |  | -0.05 (0.12) | -0.28, 0.17 |  | -0.08 (0.11) | -0.29, 0.13 |
| Agentic role nontraditionalism |  |  |  | 0.03*** (0.01) | 0.02, 0.05 |  | 0.01* (0.01) | 0.001, 0.03 |
| Gender |  |  |  | 0.68* (0.28) | 0.13, 1.23 |  | 0.60* (0.26) | 0.09, 1.10 |
| Time x Gender |  |  |  | 0.17 (0.16) | -0.15, 0.49 |  | 0.03 (0.15) | -0.26, 0.32 |
| Agentic role nontraditionalism x Gender |  |  |  | -0.03** (0.01) | -0.05, -0.01 |  | -0.01 (0.01) | -0.03, 0.01 |
| Constant | 26.45*** (0.69) | 25.10, 27.80 |  | 3.44*** (0.20) | 3.04, 3.83 |  | 3.38*** (0.18) | 3.02, 3.74 |
|  | *R*^2^ = .26 | |  | *R*^2^ = .07 | |  | *R*^2^ = 04 | |
|  | *F*(1,314) = 109.38, *p* < .001 | |  | *F*(5,310) = 4.51, *p* <.001 | |  | *F*(5,310) = 2.58, *p* = .03 | |
| Index of moderated mediation |  | |  | Index = -0.23  95% CI = -0.44, -0.06 | |  | Index = -0.11  95% CI = -0.32, 0.08 | |

*Note.*  **p*<.05, ***p*<.01, ****p*<.001

**Table C5.** Indirect effects of time on masculine cognitive dimension through agentic nontraditionalism conditional on target gender. Unstandardized regression coefficients, bias corrected bootstrapped standard errors and confidence intervals using 10 000 samples.

|  | Masculine cognitive | |
| --- | --- | --- |
|  | *b (SE)* | 95% CI |
| Women | 0.26* (0.09) | 0.13, 0.46 |
| Men | 0.03 (0.06) | -0.07, 0.16 |

*Note.* *significant indirect effect, bootstrapped CI does not include 0

**Table C6.** Study 2. Unstandardized regression coefficients (standard errors in parentheses) with confidence intervals for estimating the indirect conditional effect of time on feminine personality through agentic nontraditionalism, moderated by target gender.

|  | Agentic role nontraditionalism | |  | Feminine personality | |
| --- | --- | --- | --- | --- | --- |
| Predictors | *b* | 95% CI |  | *b* | 95% CI |
| Time | 16.49*** (1.06) | 14.41, 18.57 |  | -0.12 (0.09) | -0.29, 0.06 |
| Women in agentic roles | 1.54 (1.28) | -0.98, 4.05 |  | -0.04 (0.06) | -0.17, 0.08 |
| Men in communal roles | 0.04 (1.28) | -2.48, 2.55 |  | -0.04 (0.06) | -0.16, 0.09 |
| Agentic role nontraditionalism |  |  |  | -0.01 (0.003) | -0.01, 0.001 |
| Gender |  |  |  | -1.18*** (0.10) | -1.37, -0.99 |
| Time × Gender |  |  |  | 0.30* (0.12) | 0.06, 0.55 |
| Agentic role nontraditionalism × Gender |  |  |  | 0.004 (0.004) | -0.004, 0.01 |
| Constant | 15.68*** (1.01) | 13.70, 17.66 |  | 5.15*** (0.08) | 5.00, 5.30 |
|  | *R*^2^ = .28 | |  | *R*^2^ = .34 | |
|  | *F*(3,640) = 81.17, *p* < .001 | |  | *F*(7,636) = 46.37, *p* < .001 | |
| Index of moderated mediation |  | |  | Index = 0.07  95% CI = -0.06, 0.21 | |

*Note.*  ^†^*p* <.06, **p*<.05, ***p*<.01, ****p*<.001.

**Table C7.** Unstandardized regression coefficients (standard errors in parantheses) with confidence intervals for estimating the indirect conditional effect of time on feminine personality through communal nontraditionalismm moderated by target gender.

|  | Communal role nontraditionalism | |  | Feminine personality | |
| --- | --- | --- | --- | --- | --- |
| Predictors | *b* | 95% CI |  | *b* | 95% CI |
| Time | 15.00*** (0.87) | 13.30, 16.69 |  | -0.14 (0.09) | -0.32, 0.04 |
| Women in agentic roles | 1.41 (1.05) | -0.64, 3.47 |  | -0.04 (0.06) | -0.17, 0.08 |
| Men in communal roles | -0.56 (1.05) | -2.62, 1.49 |  | -0.03 (0.06) | -0.16, 0.09 |
| Communal role nontraditionalism |  |  |  | -0.004 (0.003) | -0.01, 0.003 |
| Gender |  |  |  | -1.20*** (0.11) | -1.42, -0.97 |
| Time × Gender |  |  |  | 0.30* (0.13) | 0.05, 0.55 |
| Communal role nontraditionalism × Gender |  |  |  | 0.005 (0.005) | -0.01, 0.01 |
| Constant | 17.28*** (0.82) | 15.66, 18.90 |  | 5.14*** (0.09) | 4.97, 5.31 |
|  | *R*^2^ = .32 | |  | *R*^2^ = .34 | |
|  | *F*(3,640) = 101.15, *p* < .001 | |  | *F*(7,636) = 45.98, *p* < .001 | |
| Index of moderated mediation |  | |  | Index = 0.07  95% CI = -0.08, 0.24 | |

*Note.*  **p*<.05, ***p*<.01, ****p*<.001
