# Supplementary material for: The (Not So) Changing Man: Dynamic Gender Stereotypes in Sweden
Source: Front Psychol. 2019 Jan 30;10:37. doi: 10.3389/fpsyg.2019.00037 (PMC6363713; doi:10.3389/fpsyg.2019.00037)
Supplement: Supplementary file 4 [file Table_4.DOCX]

**Appendix D: Prescriptive stereotype content results**

**Table D1.** Mean and standard deviation by target gender for prescriptive gender stereotype dimensions. Positive values indicate that characteristics are rated as advantageous, negative values indicate that characteristics are rated as disadvantageous, 0 indicates neither advantageous nor disadvantageous.

|  | Stereotype dimension | | | | | | | | | | | | | | |
| --- | --- | --- | --- | --- | --- | --- | --- | --- | --- | --- | --- | --- | --- | --- | --- |
|  | Masculinity | | | | | | |  | Femininity | | | | | | |
|  | Personality positive |  | Personality negative |  | Cognitive |  | Physical |  | Personality positive |  | Personality negative |  | Cognitive |  | Physical |
| Target gender and year | *M (SD)* |  | *M (SD)* |  | *M (SD)* |  | *M (SD)* |  | *M (SD)* |  | *M (SD)* |  | *M (SD)* |  | *M (SD)* |
| **Woman** |  |  |  |  |  |  |  |  |  |  |  |  |  |  |  |
| 1950 | -.28_a1_ (0.94) |  | -1.88_a1_ (0.81) |  | 0.36a_1_ (1.31) |  | -0.16_a1_ (1.20) |  | 1.33_a1_ (0.89) |  | -1.33_a1_ (0.80) |  | 0.47_a1_ (1.14) |  | 1.24_a1_ (1.15) |
| 2017 | 0.79_b1_ (0.74) |  | -1.27_b1_ (1.20) |  | 1.32_b1_ (1.32) |  | 0.14_a1_ (0.91) |  | 0.98_a1_ (0.88) |  | -1.21_a1_ (1.13) |  | 1.02_b1_ (0.87) |  | 0.66_b1_ (1.04) |
| 2090 | 0.74_b1_ (0.90) |  | -1.37_b1_ (0.87) |  | 1.32_b1_ (1.02) |  | 0.12_a1_ (0.74) |  | 1.01_a1_ (0.87) |  | -1.30_a1_ (0.98) |  | 1.08_b1_ (0.85) |  | 0.51_b1_ (1.10) |
| **Total** | 0.41 (0.99) |  | -1.52 (1.00) |  | 1.00 (1.22) |  | 0.03 (0.98) |  | 1.11 (0.89) |  | -1.28 (0.97) |  | 0.86 (1.00) |  | 0.81 (1.14) |
| **Man** |  |  |  |  |  |  |  |  |  |  |  |  |  |  |  |
| 1950 | 0.51_a2_ (1.03) |  | -1.01_a2_ (0.98) |  | 1.04_a2_ (1.32) |  | 0.87_a2_ (1.07) |  | 0.31_a2_ (1.09) |  | -1.40_a1_ (0.89) |  | 0.34_a1_ (1.16) |  | 0.08_a2_ (1.22) |
| 2017 | 0.54_a1_ (0.83) |  | -1.12_a1_ (1.14) |  | 1.52_b1_ (0.93) |  | 0.54_a2_ (0.98) |  | 0.66_a1_ (0.97) |  | -1.40_a1_ (0.77) |  | 0.66_a1_ (0.89) |  | -0.003_a2_ (0.89) |
| 2090 | 0.55_a1_ (0.94) |  | -1.27_a1_ (1.18) |  | 1.16_b1_ (1.34) |  | 0.38_ab1_ (0.91) |  | 0.54_a2_ (1.18) |  | -1.35_a1_ (1.00) |  | 0.64_a2_ (1.06) |  | 0.17_a1_ (1.25) |
| **Total** | 0.53 (0.93) |  | -1.14 (1.11) |  | 1.23 (1.23) |  | 0.58 (1.00) |  | 0.50 (1.09) |  | -1.38 (0.89) |  | 0.55 (1.05) |  | 0.09 (1.13) |

*Note.* Within each target gender, means with different column subscripts (a,b) differ significantly at *p* < .05 between time points. Within each time point, means with different column subscripts (1,2) differ significantly at *p* < .05 between women and men.

**Table D2.** Analysis of the effect of year, target gender, and year x target gender for prescriptive gender stereotype content

|  | Stereotype dimension | | | | | | | | | | | | | | | | | | | | | | |
| --- | --- | --- | --- | --- | --- | --- | --- | --- | --- | --- | --- | --- | --- | --- | --- | --- | --- | --- | --- | --- | --- | --- | --- |
|  | Masculinity | | | | | | | | | | |  | Femininity | | | | | | | | | | |
|  | Personality positive | |  | Personality negative | |  | Cognitive | |  | Physical | |  | Personality positive | |  | Personality negative | |  | Cognitive | |  | Physical | |
|  | *F* | η^2^_p_ |  | *F* | η^2^_p_ |  | *F* | η^2^_p_ |  | *F* | η^2^_p_ |  | *F* | η^2^_p_ |  | *F* | η^2^_p_ |  | *F* | η^2^_p_ |  | *F* | η^2^_p_ |
| Target year (2) | 0.07 | <0.001 |  | 0.10 | 0.001 |  | 7.01** | 0.04 |  | 2.99^†^ | 0.02 |  | 12.68*** | 0.07 |  | 1.52 | 0.01 |  | 10.73*** | 0.06 |  | 0.35 | 0.002 |
| Target gender (1) | 29.81*** | 0.09 |  | 0.98 | 0.003 |  | 7.88** | 0.02 |  | 33.56*** | 0.1 |  | 1.38 | 0.004 |  | 10.60* | 0.03 |  | 3.21 | 0.01 |  | 26.30*** | 0.08 |
| Year x Gender (2) | 3.55* | 0.02 |  | 0.18 | 0.001 |  | 0.0.72 | 0.01 |  | 3.72* | 0.02 |  | 11.02*** | 0.07 |  | 4.52* | 0.03 |  | 3.56* | 0.02 |  | 4.64* | 0.03 |
| *R^2^* | .11 | |  | .005 | |  | .068 | |  | .16 | |  | .14 | |  | .068 | |  | .093 | |  | .10 | |

*Note.* ^†^*p* <.06, **p*<.05, ***p*<.01, ****p*<.001. *df1* is presented by its corresponding factor, and *df2* = 316 for all dimensions.

**Table D3.** Unstandardized regression coefficients (standard errors in parentheses) with confidence intervals for estimating the indirect conditional effect of time on prescriptive masculine stereotype content through agentic nontraditionalism moderated by target gender.

|  | Agentic role nontraditionalism | |  | Masculine positive personality | |  | Masculine negative personality | |  | Masculine cognitive | |  | Masculine physical | |
| --- | --- | --- | --- | --- | --- | --- | --- | --- | --- | --- | --- | --- | --- | --- |
| Predictors | *b* | 95% CI |  | *b* | 95% CI |  | *b* | 95% CI |  | *b* | 95% CI |  | *b* | 95% CI |
| Time | 8.69*** (0.83) | 7.05, 10.32 |  | 0.34** (0.10) | 0.14, 0.54 |  | 0.06 (0.12) | -0.17, 0.29 |  | 0.27* (0.13) | 0.001, 0.53 |  | 0.09 (0.11) | -0.13, 0.30 |
| Agentic role nontraditionalism |  |  |  | 0.022*** (0.01) | 0.01, 0.04 |  | 0.02** (0.01) | 0.01, 0.04 |  | 0.02** (0.01) | 0.01, 0.04 |  | 0.01 (0.01) | -0.01, 0.02 |
| Gender |  |  |  | 1.00*** (0.25) | 0.52, 1.49 |  | 1.16*** (0.28) | 0.60, 1.71 |  | 0.83* (0.33) | 0.19, 1.47 |  | 1.09*** (0.26) | 0.57, 1.61 |
| Time x Gender |  |  |  | -0.20 (0.14) | -0.48, 0.10 |  | -0.09 (0.16) | -0.41, 0.24 |  | -0.21 (0.19) | -0.58, 0.16 |  | -0.22 (0.15) | -0.52, 0.08 |
| Agentic role nontraditionalism x Gender |  |  |  | -0.03*** (0.01) | -0.05, -0.02 |  | -0.03** (0.01) | -0.05, -0.01 |  | -0.03* (0.01) | -0.05, -0.003 |  | -0.02* (0.01) | -0.04, -0.001 |
| Constant | 26.45*** (0.69) | 25.10, 27.80 |  | -0.14 (0.18) | -0.49, 0.21 |  | -2.06*** (0.20) | -2.46, -1.66 |  | 0.43 (0.24) | -0.04, 0.89 |  | -0.18 (0.19) | -0.55, 0.20 |
|  | *R*^2^ = .26 | |  | *R*^2^ = .14 | |  | *R*^2^ = .08 | |  | *R*^2^ = .08 | |  | *R*^2^ = .11 | |
|  | *F*(1,314) = 109.38, *p* < .001 | |  | *F*(5,310) = 10.38, *p* < .001 | |  | *F*(5,310) = 5.28, *p* < .001 | |  | *F*(5,310) = 5.16, *p* < .001 | |  | *F*(5,310) = 8.00, *p* < .001 | |
| Index of moderated mediation |  | |  | Index = -0.30  95% CI = -0.47, -0.14 | |  | Index = -0.27  95% CI = -0.49, -0.11 | |  | Index = -0.22  95% CI = -0.43, 0.01 | |  | Index = -0.17  95% CI = -0.36, 0.02 | |

*Note.* ^†^*p* <.06, **p*<.05, ***p*<.01, ****p*<.001.

**Table D4.** Indirect effects of time through agentic nontraditionalism conditional on target gender. Unstandardized regression coefficients, bias corrected bootstrapped standard errors and confidence intervals using 10 000 samples.

|  | Masculine positive personality | |  | Masculine negative personality | |  | Masculine cognitive | |
| --- | --- | --- | --- | --- | --- | --- | --- | --- |
|  | *b (SE)* | 95% CI |  | *b (SE)* | 95% CI |  | *b (SE)* | 95% CI |
| Women | 0.19 (0.07) | 0.08, 0.35 |  | 0.20* (0.08) | 0.07, 0.37 |  | 0.21* (0.08) | 0.07, 0.40 |
| Men | -0.10* (0.05) | -0.18, -0.003 |  | -0.07 (0.06) | -0.21, 0.03 |  | -0.01 (0.08) | -0.12, 0.18 |

*Note.* *significant indirect effect, bootstrapped CI does not include 0

**Table D5.** Unstandardized regression coefficients (standard errors in parentheses) with confidence intervals for estimating the indirect conditional effect of time on prescriptive masculine stereotype content through communal nontraditionalism moderated by target gender.

|  | Communal role nontraditionalism | |  | Masculine positive personality | |  | Masculine negative personality | |  | Masculine cognitive | |  | Masculine physical | |
| --- | --- | --- | --- | --- | --- | --- | --- | --- | --- | --- | --- | --- | --- | --- |
| Predictors | *b* | 95% CI |  | *b* | 95% CI |  | *b* | 95% CI |  | *b* | 95% CI |  | *b* | 95% CI |
| Time | 10.37*** (0.76) | 8.86, 11.87 |  | 0.40*** (0.12) | 0.17, 0.63 |  | 0.18 (0.14) | -0.09, 1.12 |  | 0.38* (0.16) | 0.08, 0.69 |  | 0.16 (0.13) | -0.09, 0.41 |
| Communal role nontraditionalism |  |  |  | 0.01 (0.01) | -0.003, 0.03 |  | 0.01 (0.01) | -0.01, 0.03 |  | 0.01 (0.01) | -0.01, 0.03 |  | -0.001 (0.01) | -0.02, 0.01 |
| Gender |  |  |  | 0.26 (0.27) | -0.28, 0.79 |  | 0.50 (0.31) | -0.11, 1.12 |  | -0.73 (0.35) | -0.77, 0.62 |  | 0.52 (0.29) | -0.05, 1.09 |
| Time x Gender |  |  |  | -0.44** (0.16) | -0.75, -0.13 |  | -0.34^†^ (0.18) | -0.69, 0.02 |  | -0.52* (0.21) | -0.92, -0.12 |  | -0.40* (0.17) | -0.74, -0.07 |
| Communal role nontraditionalism x Gender |  |  |  | -0.01 (0.01) | -0.02, 0.01 |  | -0.01 (0.01) | -0.03, 0.02 |  | 0.01 (0.01) | -0.02, 0.03 |  | 0.002 (0.01) | -0.02, 0.02 |
| Constant | 27.04*** (0.63) | 25.80, 28.29 |  | 0.11 (0.20) | -0.28, 0.51 |  | -1.73*** (0.23) | -2.18, -1.28 |  | 0.77** (0.26) | 0.26, 1.28 |  | 0.06 (0.22) | -0.36, 0.48 |
|  | *R*^2^ = .37 | |  | *R*^2^ = .11 | |  | *R*^2^ = .06 | |  | *R*^2^ = .082 | |  | *R*^2^ = .10 | |
|  | *F*(1,319) = 184.09, *p* < .001 | |  | *F*(5,315) = 8.08, *p* < .001 | |  | *F*(5,315) = 4.15, *p* = .0011 | |  | *F*(5,315) = 5.61, *p* < .001 | |  | *F*(5,315) = 6.92, *p* < .001 | |
| Index of moderated mediation |  | |  | Index = -0.06  95% CI = -0.24, 0.16 | |  | Index = -0.05  95% CI = -0.29, 0.16 | |  | Index = 0.10  95% CI = -0.14, 0.44 | |  | Index = 0.02  95% CI = -0.19, 0.26 | |

*Note.* ^†^*p* <.06, **p*<.05, ***p*<.01, ****p*<.001

**Table D6.** Unstandardized regression coefficients (standard errors in parentheses) with confidence intervals for estimating the indirect conditional effect of time on prescriptive feminine stereotype content through communal nontraditionalism moderated by target gender.

|  | Communal role nontraditionalism | |  | Feminine positive personality | |  | Feminine negative personality | |  | Feminine cognitive | |  | Feminine physical | |
| --- | --- | --- | --- | --- | --- | --- | --- | --- | --- | --- | --- | --- | --- | --- |
| Predictors | *b* | 95% CI |  | *b* | 95% CI |  | *b* | 95% CI |  | *b* | 95% CI |  | *b* | 95% CI |
| Time | 10.37*** (0.76) | 8.86, 11.87 |  | -0.12 (0.13) | -0.37, 0.13 |  | 0.01 (0.12) | -0.23, 0.25 |  | 0.28* (0.13) | 0.03, 0.54 |  | -0.30* (0.15) | -0.58, -0.01 |
| Communal role nontraditionalism |  |  |  | -0.004 (0.01) | -0.02, 0.01 |  | 0.002 (0.01) | -0.01, 0.02 |  | 0.002 (0.01) | -0.01, 0.02 |  | -0.01 (0.01) | -0.03, 0.01 |
| Gender |  |  |  | -1.05*** (0.29) | -1.62, -0.47 |  | -0.23 (0.28) | -0.77, 0.32 |  | -0.73* (0.30) | -1.32, -0.15 |  | -0.98** (0.33) | -1.63, -0.32 |
| Time x Gender |  |  |  | 0.12 (0.17) | -0.22, 0.46 |  | -0.05 (0.16) | -0.36, 0.27 |  | -0.30 (0.17) | -0.63, 0.05 |  | 0.33 (0.19) | -0.06, 0.71 |
| Communal role nontraditionalism x Gender |  |  |  | 0.02 (0.01) | -0.01, 0.04 |  | 0.004 (0.01) | -0.02, 0.02 |  | 0.01 (0.01) | -0.01, 0.03 |  | 0.01 (0.012) | -0.01, 0.03 |
| Constant | 27.04*** (0.63) | 25.80, 28.29 |  | 1.23*** (0.22) | 0.80, 1.66 |  | -1.33*** (0.20) | -1.74, -0.93 |  | 0.81*** (0.22) | 0.38, 1.25 |  | 0.99*** (0.25) | 0.51, 1.48 |
|  | *R*^2^ = .37 | |  | *R*^2^ = .11 | |  | *R*^2^ = .01 | |  | *R*^2^ = .08 | |  | *R*^2^ = .13 | |
|  | *F*(1,319) = 184.09, *p* < .001 | |  | *F*(5,315) = 7.69, *p* < .001 | |  | *F*(5,315) = 0.41, *p* = .84 | |  | *F*(5,315) = 5.27, *p* <.001 | |  | *F*(5,315) = 9.38, *p* < .001 | |
| Index of moderated mediation |  | |  | Index = 0.16  95% CI = -0.04, 0.42 | |  | Index = 0.05  95% CI = -0.21, 0.25 | |  | Index = 0.15  95% CI = -0.06, 0.41 | |  | Index = 0.10  95% CI = -0.14, 0.36 | |

*Note.* ^†^*p* <.06, **p*<.05, ***p*<.01, ****p*<.001

**Table D7.** Unstandardized regression coefficients (standard errors in parentheses) with confidence intervals for estimating the indirect conditional effect of time on prescriptive feminine stereotype content through agentic nontraditionalism moderated by target gender.

|  | Agentic role nontraditionalism | |  | Feminine positive personality | |  | Feminine negative personality | |  | Feminine cognitive | |  | Feminine physical | |
| --- | --- | --- | --- | --- | --- | --- | --- | --- | --- | --- | --- | --- | --- | --- |
| Predictors | *b* | 95% CI |  | *b* | 95% CI |  | *b* | 95% CI |  | *b* | 95% CI |  | *b* | 95% CI |
| Time | 8.69*** (0.83) | 7.05, 10.32 |  | -0.15 (0.11) | -0.37, 0.07 |  | -0.15 (0.10) | -0.36, 0.05 |  | 0.22 (0.11) | -0.003, 0.45 |  | -0.40** (0.13) | -0.65, -0.15 |
| Agentic role nontraditionalism |  |  |  | -0.001 (0.01) | -0.02, 0.01 |  | 0.02** (0.01) | 0.01, 0.03 |  | 0.01 (0.01) | -0.004, 0.03 |  | 0.01 (0.01) | -0.01, 0.02 |
| Gender |  |  |  | -0.72** (0.27) | -1.26, -0.18 |  | 0.31 (0.25) | -0.18, 0.81 |  | -0.12 (0.28) | -0.67, 0.42 |  | -0.40 (0.31) | -1.01, 0.21 |
| Time x Gender |  |  |  | 0.24 (0.16) | -0.07, 0.55 |  | 0.20 (0.15) | -0.08, 0.49 |  | -0.09 (0.16) | -0.40, 0.23 |  | 0.51** (0.18) | 0.16, 0.86 |
| Agentic role nontraditionalism x Gender |  |  |  | 0.004 (0.01) | -0.02, 0.02 |  | -0.02* (0.01) | -0.04, -0.001 |  | -0.01 (0.01) | -0.3, 0.01 |  | -0.01 (0.01) | -0.03, 0.01 |
| Constant | 26.45*** (0.69) | 25.10, 27.80 |  | 1.13*** (0.20) | 0.74, 1.52 |  | -1.75*** (0.18) | -2.11, -1.40 |  | 0.59** (0.20) | 0.20, 0.99 |  | 0.67** (0.22) | 0.23, 1.10 |
|  | *R*^2^ = .26 | |  | *R*^2^ = .10 | |  | *R*^2^ = .03 | |  | *R*^2^ = .07 | |  | *R*^2^ = .12 | |
|  | *F*(1,314) = 109.38, *p* < .001 | |  | *F*(5,310) = 6.72, *p* < .001 | |  | *F*(5,310) = 2.13, *p* = .062 | |  | *F*(5,310) = 4.54, *p* <.001 | |  | *F*(5,310) = 8.72, *p* < .001 | |
| Index of moderated mediation |  | |  | Index = 0.03  95% CI = -0.13, 0.24 | |  | Index = -0.16  95% CI = -0.34, 0.004 | |  | Index = -0.08  95% CI = -0.27, 0.12 | |  | Index = -0.10  95% CI = -0.27, 0.08 | |

*Note.* ^†^*p* <.06, **p*<.05, ***p*<.01, ****p*<.001
